# Schizophrenia-associated SAP97 mutations increase glutamatergic synapse strength in the dentate gyrus and impair contextual episodic memory in rats

**DOI:** 10.1038/s41467-022-28430-5

**Published:** 2022-02-10

**Authors:** Yuni Kay, Linda Tsan, Elizabeth A. Davis, Chen Tian, Léa Décarie-Spain, Anastasiia Sadybekov, Anna N. Pushkin, Vsevolod Katritch, Scott E. Kanoski, Bruce E. Herring

**Affiliations:** 1 Neuroscience Graduate Program, University of Southern California, Los Angeles, CA 90089 USA; 2 Department of Biological Sciences, Human and Evolutionary Biology Section, Dornsife College of Letters, Arts and Sciences, University of Southern California, Los Angeles, CA 90089 USA; 3 Department of Chemistry, University of Southern California, Los Angeles, CA 90089 USA; 4 Quantitative and Computational Biology, University of Southern California, Los Angeles, CA 90089 USA; 5 Department of Biological Sciences, Neurobiology Section, Dornsife College of Letters, Arts and Sciences, University of Southern California, Los Angeles, CA 90089 USA

**Keywords:** Schizophrenia, Molecular neuroscience, Neurotransmitters

## Abstract

Mutations in the putative glutamatergic synapse scaffolding protein SAP97 are associated with the development of schizophrenia in humans. However, the role of SAP97 in synaptic regulation is unclear. Here we show that SAP97 is expressed in the dendrites of granule neurons in the dentate gyrus but not in the dendrites of other hippocampal neurons. Schizophrenia-related perturbations of SAP97 did not affect CA1 pyramidal neuron synapse function. Conversely, these perturbations produce dramatic augmentation of glutamatergic neurotransmission in granule neurons that can be attributed to a release of perisynaptic GluA1-containing AMPA receptors into the postsynaptic densities of perforant pathway synapses. Furthermore, inhibiting SAP97 function in the dentate gyrus was sufficient to impair contextual episodic memory. Together, our results identify a cell-type-specific synaptic regulatory mechanism in the dentate gyrus that, when disrupted, impairs contextual information processing in rats.

## Abstract (web summary)

The effects of SAP97 mutations associated with schizophrenia on synaptic function are unclear. Here, the authors show that schizophrenia-related SAP97 mutations enhance glutamatergic synapse strength in the dentate gyrus, impairing contextual episodic memory in rats.

## Introduction

Schizophrenia is a debilitating psychiatric disorder that affects around 20 million people worldwide. Symptoms of this disorder include hallucinations, delusions, flat affect, the loss of a sense of personal identity, poor executive function, and deficits in memory^1,2^. Despite many years of research, it remains unclear which cellular and molecular mechanisms in the brain are disrupted in individuals with schizophrenia. It is also unclear which specific regions in the brain are strongly affected by the disruption of these mechanisms. Accumulating evidence supports a connection between SAP97 loss of function and schizophrenia^3–14^. For example: the gene encoding SAP97, *DLG1*, was recently identified as a potential hub of schizophrenia-related synaptic dysfunction^13^; missense mutations in SAP97 have been identified in individuals with schizophrenia^12,14^; and microdeletion mutations in humans resulting in the loss of a *DLG1* allele give rise to a 40-fold increase in the risk of developing schizophrenia^8,9^. SAP97 is a Membrane-Associated Guanylate Kinase (MAGUK) protein. MAGUK protein family members include PSD-95, PSD-93, SAP102, and SAP97. PSD-95, PSD-93, and SAP102 are major constituents of the glutamatergic synapse postsynaptic density (PSD). At the postsynaptic side of glutamatergic synapses, PSD-95, PSD-93, and SAP102 provide scaffolds for synaptic protein complexes via their three PSD-95/Dlg/ZO1 (PDZ) domains^15^. It is well established that PSD-95, PSD-93, and SAP102 use their PDZ domains to anchor AMPA and NMDA receptors (AMPARs and NMDARs) in the PSD in direct opposition to regions of presynaptic glutamate release. This MAGUK protein-mediated glutamate receptor positioning is critical for efficient activation of these receptors by presynaptically-released glutamate.

In contrast to other MAGUK proteins, the role of SAP97 in synaptic regulation is uncertain, despite the fact that SAP97 is the only MAGUK that can interact directly with AMPARs. This interaction occurs through SAP97’s PDZ2 domain binding to the C-terminal PDZ-binding domain of the AMPAR subunit GluA1^16–20^. It is also unclear whether the C-tail of GluA1 plays any role in synaptic regulation^18,21^. Previous studies have provided conflicting data regarding SAP97’s influence on synaptic function^22–27^. Most of these studies have relied on recombinant expression of SAP97 and/or dissociated neuron cultures where synapses form between unknown neuron subtypes. However, one particularly notable study has shown that knocking out SAP97 led to no alteration in glutamatergic neurotransmission in hippocampal CA1 pyramidal neurons^28^. Additionally, mutant mice expressing a PDZ-binding domain lacking the form of the GluA1 subunit which prevents AMPARs from binding to SAP97 (GluA1-Δ7 mice) were found to have normal glutamatergic neurotransmission in hippocampal CA1 pyramidal neurons^18^. Together, these studies have been used to support a compelling argument against a role for SAP97 in the regulation of glutamatergic synaptic transmission^15^. This lack of understanding regarding SAP97’s role in the brain has been particularly frustrating given SAP97’s growing implication in schizophrenia, SAP97’s similarity to other essential synaptic proteins, and the fact that schizophrenia is largely considered to be a synaptic disease^13,14,29^.

Increasing evidence points to dysfunction of the dentate gyrus as a contributing factor in the development of schizophrenia^1,30–38^. The dentate gyrus serves as the gateway for information coming into the hippocampus via the perforant pathway, and symptoms associated with schizophrenia exhibit a high degree of correlation with symptoms stemming from dentate gyral dysfunction^1,30,39^. In the present study, we find that the expression of βSAP97, the major isoform of SAP97^24^, is absent in the apical and basal dendrites of CA3 and CA1 pyramidal neurons of the hippocampus. In contrast, robust βSAP97 expression is observed in the dendrites of granule neurons in the dentate gyrus (DG granule neurons). Given that reduced SAP97 expression in the brain is associated with a substantially increased risk of developing schizophrenia^3,8,9^, we were interested in whether βSAP97 regulates glutamatergic synapse function in DG granule neurons and whether reduced βSAP97 expression in the dentate gyrus is sufficient to produce schizophrenia-related behavioral phenotypes in rodents.

Here we find that reducing βSAP97 expression in DG granule neurons results in a dramatic increase in AMPAR-mediated synaptic transmission following perforant path stimulation. Conversely, inhibition of βSAP97 expression in CA1 pyramidal neurons had no effect on glutamatergic synaptic transmission. Furthermore, we find that reducing βSAP97 expression specifically within the dentate gyrus is sufficient to disrupt contextual episodic memory processing in rats. Similar memory deficits are present in individuals with schizophrenia and have been proposed to contribute to the development of delusions, disorganization, hallucinations, and the loss of a sense of personal identity observed with this disorder^40–52^. Finally, we show that schizophrenia-related missense mutations clustered in SAP97’s PDZ2 domain also produce large increases in synaptic AMPAR function in DG granule neurons that can be attributed to the release of perisynaptic GluA1-containing AMPARs into the PSDs of perforant pathway synapses. Altogether, our study identifies a cell-type-specific synaptic regulatory mechanism in the dentate gyrus that, when disrupted, impairs contextual episodic memory in rats.

## Results

### βSAP97 knockdown augments synaptic AMPAR-mediated neurotransmission in DG granule neurons

**Fig. 1:**
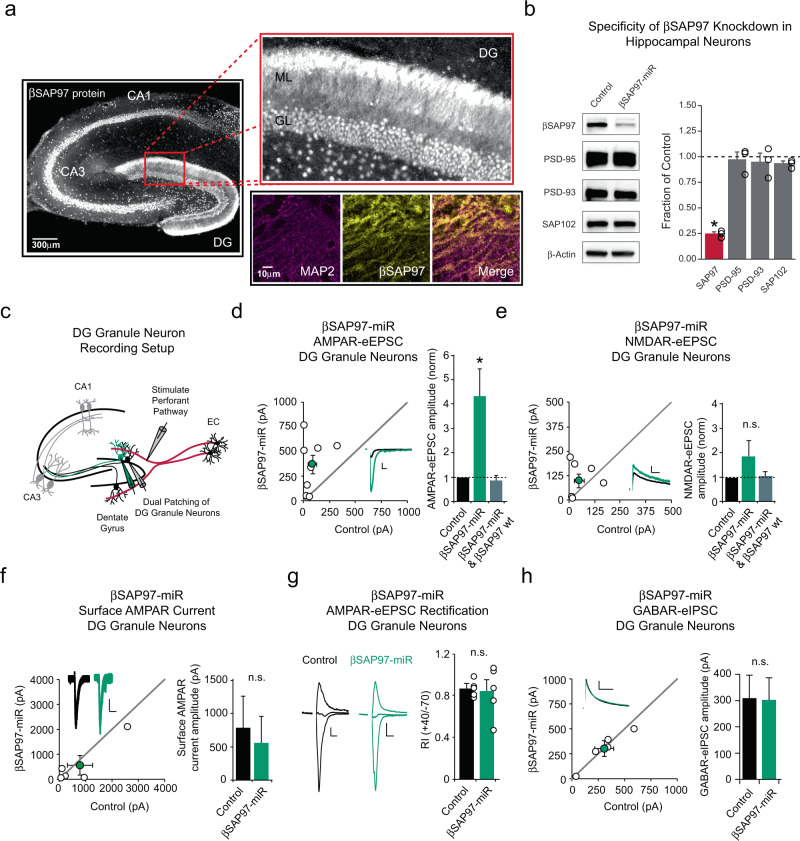
βSAP97 knockdown augments AMPAR-mediated neurotransmission in DG granule neurons. **a** (Left) Representative immunolabeling of βSAP97 in a rat entorhino-hippocampal slice. Red box shows enlarged portion of the dentate gyrus. GL, granule layer; ML, molecular layer. (Right) Co-immunolabeling of MAP2 and βSAP97 in dendrites of DG granule neurons. **b** Western blot analysis showing specificity of βSAP97-miR in dissociated hippocampal neurons. βSAP97-miR reduces βSAP97 protein expression in dissociated hippocampal neurons without altering PSD-95, PSD-93, and SAP102 protein expression. (Right) Bar graph shows quantification of SAP97, PSD-95, PSD-93, and SAP102 protein expression following βSAP97-miR-mediated knockdown. n represents independent experiments. (βSAP97, *n* = 4, *p* = 0.00002; PSD-95, *n* = 3, *p* = 0.75; PSD-93, *n* = 3, *p* = 0.62; SAP102, *n* = 3, *p* = 0.09; two sample *T* tests). **c** Schematic representation of electrophysiological recording setup for DG granule neurons. For panels (**d**) and (**e**), open circles are single pairs of control and transfected neurons, filled circles represent the mean amplitudes (±SEM), insets show representative current traces from control (black) and βSAP97-miR transfected (green) neurons with stimulation artifacts removed. Scale bars: 20 ms for AMPA, 50 ms for NMDA, 100pA. Bar graphs show the average AMPAR-eEPSC and NMDAR-eEPSC amplitudes (±SEM) of DG granule neurons expressing the βSAP97-miR (green) and DG granule neurons co-expressing the βSAP97-miR and miR-resistant wild-type (wt) βSAP97 cDNA (gray) normalized to their respective control cell average eEPSC amplitudes (black). βSAP97-miR expression increases AMPAR-eEPSC amplitude in DG granule neurons (*n* = 8 pairs, *p* = 0.019, paired *T* test) (**d**) but has no detectable effect on NMDAR-eEPSC amplitude (*n* = 7 pairs, *p* = 0.27, paired *T* test) (**e**). Co-expression of βSAP97-miR and miR-resistant βSAP97 cDNA has no detectable effects on either AMPAR-eEPSC (*n* = 8 pairs, *p* = 0.227, paired *T* test) (**d**) or NMDAR-eEPSC amplitudes (*n* = 8 pairs, *p* = 0.99, paired *T* test) (**e**). Additional details of the rescue experiment are provided in Supplementary Fig. 2b, c, e. **f** βSAP97-miR expression does not change surface AMPAR current amplitude in DG granule neurons. (Left) Open circles in the scatter plot represent single pairs of control and transfected neurons, filled circles represent the mean amplitudes (±SEM), insets show representative surface AMPAR current traces from control (black) and transfected (green) neurons. Scale bars: 5 s, 25pA. (Right) Bar graph shows average surface AMPAR current amplitudes (±SEM) of control (black) and βSAP97-miR expressing (green) DG granule neurons (*n* = 5 pairs, *p* = 0.335, paired *T* test). **g** βSAP97 knockdown does not change AMPAR-eEPSC rectification. (Left) Representative current traces, scale bars: 20 ms, 20 pA. (Right) Bar graph shows mean ± SEM of the AMPAR-eEPSC rectification index of control (black, *n* = 5 neurons) and βSAP97-miR expressing (green, *n* = 5 neurons) DG granule neurons (*p* = 0.81, two sample *T* test). **h** βSAP97 knockdown does not affect GABAergic synapse function in DG granule neurons. Open circles are single pairs of control and transfected neurons, filled circles represent the mean amplitudes (±SEM), insets show representative current traces from control (black) and βSAP97-miR transfected (green) neurons with stimulation artifacts removed. Scale bars: 100pA, 100 ms. Bar graph shows the average GABAR-eIPSC amplitudes (±SEM) of DG granule neurons expressing the βSAP97-miR (green) and control DG neurons (black) (*n* = 5 pairs, p = 0.81, paired *T* test). *p < 0.05; n.s., not significant. All statistical tests performed were two-tailed. Source data are provided in the Source Data file.

Both dentate gyral and SAP97 dysfunction have been implicated in the development of schizophrenia. To determine where βSAP97 is endogenously expressed in the rat hippocampus, we performed an immunohistochemical analysis of βSAP97 expression in hippocampal slices from rats. Remarkably, this analysis revealed robust expression of βSAP97 in the molecular layer of the dentate gyrus but not in the stratum radiatum or stratum oriens of CA3 and CA1 (Fig. 1a). βSAP97 expression overlapped with the dendritic-marker MAP2 in the molecular layer of the dentate gyrus, indicating that βSAP97 is expressed in the dendrites of DG granule neurons (Fig. 1a and Supplementary Fig. 1a). We validated this experiment using a second SAP97 antibody, which again displayed dendritic labeling that was specific to DG granule neurons (Supplementary Fig. 1b). An isotype control primary antibody, as well as the secondary antibody alone, produced no visible signal in our slices (Supplementary Fig. 1c). An immunizing peptide blocking experiment was also performed to further validate the specificity of our immunolabeling, and again no visible signal was found with the peptide blocked sample, demonstrating that the immunolabeling was specific (Supplementary Fig. 1c). Together, these results suggest that glutamatergic perforant pathway synapses between presynaptic entorhinal cortical neurons and postsynaptic DG granule neurons may be selectively regulated by βSAP97 in DG granule neurons. To determine whether βSAP97 plays a role in DG granule neuron glutamatergic synapse regulation, we generated an RNAi against βSAP97. AAV-mediated expression of our βSAP97-microRNA (miR) construct in dissociated rat hippocampal neurons reduced endogenous βSAP97 protein levels by ~75% with no effects observed on the expression of other MAGUK proteins (Fig. 1b). Using our miR construct, we knocked down the expression of βSAP97 in DG granule neurons in rat organotypic entorhino-hippocampal slice cultures through biolistic transfection^23,53^. Biolistic transfection of these slices allows for sparse transfection of neurons maintained within their intact, endogenous circuitry^54–59^. Six days after transfection, recordings of AMPA receptor- and NMDA receptor-evoked excitatory postsynaptic currents (AMPAR- and NMDAR-eEPSCs) were made from fluorescent βSAP97-miR transfected DG granule neurons and neighboring control neurons simultaneously during perforant pathway stimulation (Fig. 1c). This approach allows for a pair-wise, internally controlled comparison of the consequences of acute genetic manipulations that are limited to the postsynaptic neuron^54–59^. Knocking down βSAP97 in DG granule neurons using this approach led to a striking 4-fold increase in AMPAR-eEPSC amplitudes compared to paired control neurons (Fig. 1d), a surprising phenotype given that knocking down traditional MAGUK proteins reduces AMPAR-eEPSC amplitudes in CA1 pyramidal neurons^60^. A significant change in NMDAR-eEPSC amplitude was not observed following βSAP97 knockdown in DG granule neurons (Fig. 1e). RT-PCR analysis confirmed that the increase in AMPAR-eEPSC amplitude we observed was not due to a secondary upregulation of αSAP97 expression (Supplementary Fig. 2a). αSAP97 protein is not endogenously expressed in the hippocampus^24^ but has been shown to augment synaptic function when exogenously expressed in neurons^61^. To further verify that the 4-fold increase in AMPAR-eESPC amplitude was indeed due to knockdown of βSAP97 specifically, we generated a recombinant, RNAi-resistant βSAP97 expression construct (Supplementary Fig. 2b). We first verified that the expression of this RNAi-resistant βSAP97 was not inhibited by our βSAP97-miR (Supplementary Fig. 2c). Next, we co-expressed our βSAP97-miR with an mCherry-tagged RNAi-resistant βSAP97, and verified via imaging that this recombinant βSAP97 localized to dendritic spines, consistent with previous work demonstrating perisynaptic localization of βSAP97^61^ (Supplementary Fig. 2d). Finally, we co-expressed our βSAP97-miR and the RNAi-resistant βSAP97 construct in DG granule neurons and found that expression of the RNAi-resistant βSAP97 returned AMPAR-eEPSC amplitude to wild-type levels (Fig. 1d and Supplementary Fig. 2e), confirming the specificity of our genetic manipulation.

The increase in synaptic AMPAR-eEPSC amplitude we observe following knockdown of βSAP97 may arise from increased exocytosis of intracellular AMPARs or rearrangements of AMPARs already on surface of neurons that result in more AMPARs in the PSD of glutamatergic synapses. To determine whether βSAP97 supports intracellular AMPAR stores or inhibits surface AMPARs from entering the PSD, we puffed glutamate onto the dendrites of neighboring whole-cell patch clamped control and βSAP97-miR-expressing DG granule neurons. Puffing glutamate onto the dendrites of these neurons produced currents stemming from the activation of extrasynaptic, perisynaptic, as well as synaptic AMPARs on the surface of dendrites. This approach allowed us to compare the number of AMPARs on the dendritic surface of neurons in the presence and absence of βSAP97. We found that knocking down βSAP97 produced no change in AMPAR surface current amplitude in DG granule neurons (Fig. 1f). These data are consistent with that of previous work demonstrating that recombinant βSAP97 restricts the ability of perisynaptic AMPARs on the spine surface from accessing the PSD of glutamatergic synapses^61^.

Given that βSAP97 likely prevents a subpopulation of AMPARs from reaching synapses, we were interested in whether βSAP97 associates with AMPARs that have a subunit composition distinct from synaptic AMPARs. Previous studies have reported the presence of calcium-permeable GluA2 subunit-lacking AMPARs in neurons that are made available to synapses during activity-dependent synaptic potentiation^62^. One possibility is that βSAP97 binds to and holds GluA2-lacking AMPARs away from glutamatergic synapses in DG granule neurons, and that knocking down βSAP97 releases these receptors, allowing them to reach synapses. To test this hypothesis, we compared synaptic AMPAR rectification in control neurons to neurons expressing our βSAP97-miR. GluA2-lacking AMPARs exhibit inwardly rectifying currents whereas GluA2-containing AMPARs have a linear current/voltage relationship. We found that knocking down βSAP97 had no effect on +40/−70 AMPAR-eEPSC amplitude ratios, with both control neurons and βSAP97 knockdown neurons exhibiting linear +40/−70 mV AMPAR-eEPSC amplitude ratios characteristic of GluA2-containing AMPARs (Fig. 1g). Thus, we conclude that knocking down βSAP97 does not result in the insertion of GluA2-lacking AMPARs into glutamatergic synapses.

Finally, we performed additional basic characterizations of the effects of βSAP97 knockdown on other neuronal and synaptic properties. We determined that knockdown of βSAP97 has no effect on the resting membrane potential or the excitability of DG granule neurons (Supplementary Fig. 2f). We also found that βSAP97 knockdown in DG granule neurons has no effect on GABAergic synapse function (Fig. 1h).

### βSAP97 knockdown has no effect on glutamatergic neurotransmission in CA1 pyramidal neurons

**Fig. 2:**
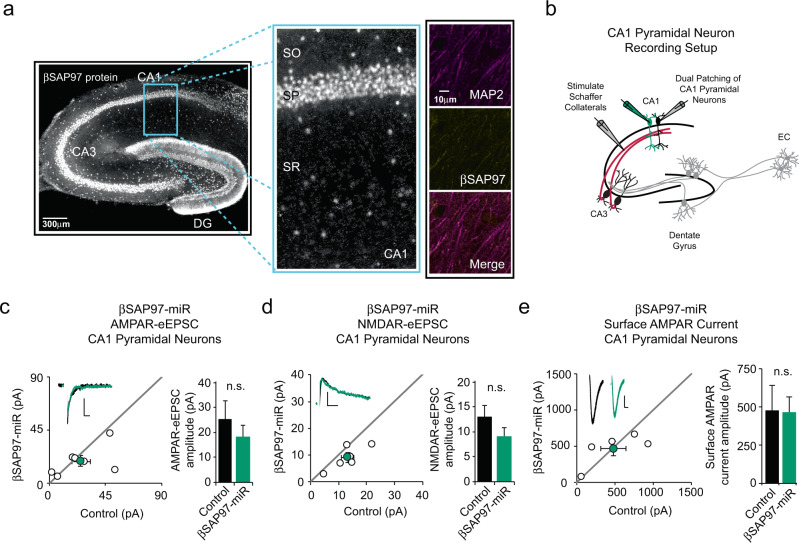
βSAP97 knockdown has no effect on glutamatergic neurotransmission in CA1 pyramidal neurons. **a** (Left) Representative immunolabeling of βSAP97 in a rat entorhino-hippocampal slice. Blue box shows enlarged CA1 region. SO, stratum oriens; SP, stratum pyramidale; SR, stratum radiatum. (Right) Co-immunolabeling of MAP2 and βSAP97 in dendrites of CA1 neurons. **b** Schematic representation of electrophysiological recording setup for CA1 pyramidal neurons. For panels (**c**) and (**d**), open circles are single pairs of control and transfected neurons, filled circles represent the mean amplitudes (±SEM), insets show representative current traces from control (black) and transfected (green) neurons with stimulation artifacts removed. Scale bars: 20 ms for AMPA, 50 ms for NMDA, 20 pA. Bar graphs show the average AMPAR-eEPSC and NMDAR-eEPSC amplitudes (±SEM) of CA1 pyramidal neurons expressing the βSAP97-miR (green) and control cell average eEPSC amplitudes (black). βSAP97-miR expression has no detectable effects on either AMPAR-eEPSC (*n* = 7 pairs, *p* = 0.29, paired *T* test, n.s., not significant) (**c**) or NMDAR-eEPSC amplitudes (*n* = 6 pairs, *p* = 0.29, paired *T* test, n.s., not significant) (**d**) in CA1 pyramidal neurons. **e** βSAP97-miR does not change surface AMPAR current amplitude in CA1 pyramidal neurons. (Left) Open circles in the scatter plot represent single pairs of control and transfected neurons, filled circles represent the mean amplitudes (±SEM), insets show representative surface AMPAR current traces from control (black) and transfected (green) neurons. Scale bars: 5 s, 200pA. (Right) Bar graph shows average surface AMPAR current amplitudes (±SEM) of control (black) and βSAP97-miR expressing (green) CA1 pyramidal neurons (*n* = 5 pairs, *p* = 0.92, paired *T* test). n.s., not significant. All statistical tests performed were two-tailed. Source data are provided in the Source Data file.

Our immunohistochemical analysis revealed no expression of βSAP97 in the dendrites of CA1 pyramidal neurons (Fig. 2a and Supplementary Fig. 1b). However, βSAP97 expression was observed in the cell bodies of these neurons. To determine whether inhibiting βSAP97 expression affects glutamatergic synaptic transmission in CA1 pyramidal neurons, we transfected these neurons with our βSAP97-miR. We then examined AMPAR- and NMDAR-eEPSC amplitudes in pairs of βSAP97-miR transfected and control CA1 pyramidal neurons following Schaffer collateral stimulation (Fig. 2b). As suspected by the lack of βSAP97 expression in the dendrites of these neurons, knocking down βSAP97 led to no significant effects on either AMPAR- or NMDAR-eEPSC amplitudes (Fig. 2c, d). We also assessed the effects of βSAP97 knockdown on dendritic AMPAR surface current in CA1 pyramidal neurons by puffing glutamate onto the apical dendrites of neighboring whole-cell patch clamped control and βSAP97-miR-expressing CA1 pyramidal neurons. We found that βSAP97 knockdown does not affect dendritic AMPAR surface current in CA1 pyramidal neurons (Fig. 2e). In further characterizing the effects of βSAP97 knockdown in CA1 pyramidal neurons, we stimulated axons within the stratum oriens to evaluate the function of glutamatergic synapses on basal dendrites (Supplementary Fig. 3a) and again found no effect on either AMPAR- or NMDAR-eEPSC amplitudes (Supplementary Fig. 3b, c). Taken together with our results in DG neurons, we have now identified a specific set of glutamatergic synapses where βSAP97 plays an essential, cell-type-specific role in regulating glutamatergic neurotransmission.

### PSD-95, PSD-93, SAP102 knockdown similarly affects CA1 pyramidal neurons and DG granule neurons

**Fig. 3:**
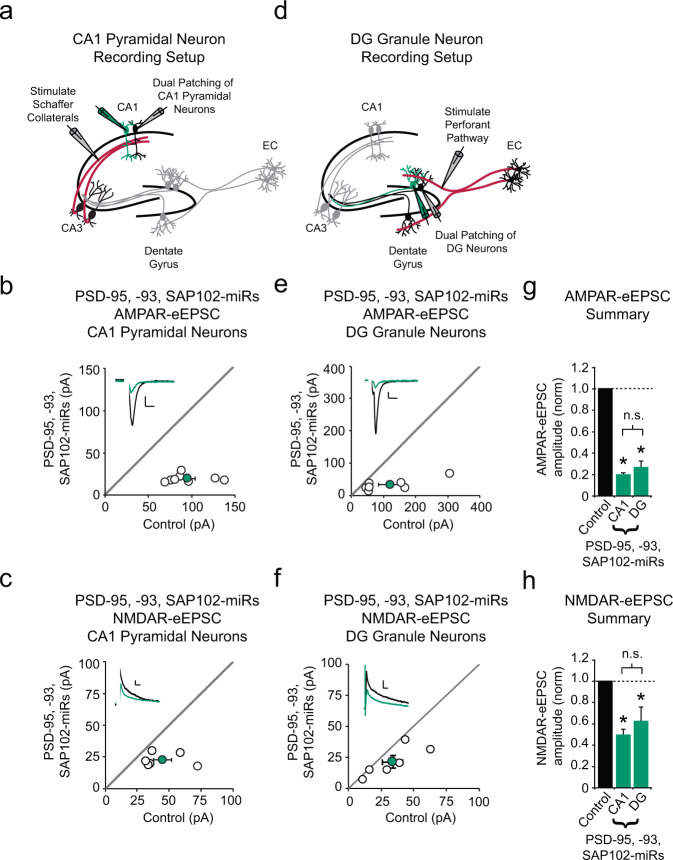
PSD-95, PSD-93, SAP102 knockdown similarly affects CA1 pyramidal neurons and DG granule neurons. **a** Schematic representation of electrophysiological recording setup for CA1 pyramidal neurons. For panels (**b**), (**c**), (**e**), and (**f**), open circles are single pairs of control and transfected neurons, filled circles represent the mean amplitudes (±SEM), insets show representative current traces from control (black) and transfected (green) neurons with stimulation artifacts removed. Scale bars: 20 ms for AMPA, 50 ms for NMDA, 20 pA. **b** PSD-95, PSD-93, and SAP102 knockdown significantly decreases AMPAR-eEPSC amplitude in CA1 pyramidal neurons (*n* = 8 pairs, *p* = 0.00006, paired *T* test). **c** PSD-95, PSD-93, and SAP102 knockdown significantly decreases NMDAR-eEPSC amplitude in CA1 pyramidal neurons (*n* = 6 pairs, *p* = 0.03, paired *T* test). **d** Schematic representation of electrophysiological recording setup for DG granule neurons. **e** PSD-95, PSD-93, and SAP102 knockdown significantly decreases AMPAR-eEPSC amplitude in DG granule neurons (*n* = 7 pairs, *p* = 0.03, paired *T* test). **f** PSD-95, PSD-93, and SAP102 knockdown significantly decreases NMDAR-eEPSC amplitude in DG granule neurons (*n* = 6 pairs, *p* = 0.045, paired *T* test). **g**, **h** Summary bar graphs show the average AMPAR-eEPSC amplitudes (±SEM) (**g**) and NMDAR-eEPSC amplitudes (±SEM) (**h**) of CA1 pyramidal neurons and DG granule neurons expressing PSD-95, PSD-93, SAP102 miRs (green) normalized to their respective control cell average eEPSC amplitudes (black). Two-sample *T* tests were used to compare across independent conditions (CA1 vs. DG); (CA1 vs. DG (AMPA), *n* = 8 CA1 pairs, 7 DG pairs, *p* = 0.12; CA1 vs. DG (NMDA), *n* = 6 CA1 pairs, 6 DG pairs, *p* = 0.34). *p < 0.05; n.s., not significant. All statistical tests performed were two-tailed. Source data are provided in the Source Data file.

In the hippocampus, our results demonstrate that βSAP97 inhibits synaptic AMPAR function specifically in DG granule neurons. This is in contrast to PSD-95, PSD-93, and SAP102, which are expressed in the dendrites of CA1 and CA3 pyramidal neurons and facilitate synaptic AMPAR and NMDAR function in CA1 pyramidal neurons and many other neurons in the brain^15,60,63–68^. In addition to being expressed in the dendrites of CA1 and CA3 pyramidal neurons, PSD-95, PSD-93, and SAP102 immunolabeling are also present in the molecular layer of the dentate gyrus^66–68^. Therefore, we considered the possibility that all major MAGUK proteins play fundamentally different roles in DG granule neurons compared to what has been conventionally established in CA1 pyramidal neurons. If this was the case, it would suggest that the phenotype we observed in DG granule neurons following βSAP97 knockdown is due to general MAGUK function being different in DG granule neurons, rather than βSAP97 itself playing a unique regulatory role at perforant path-DG granule neuron synapses. To test this, we compared the result of knocking down PSD-95, PSD-93, and SAP102 in either CA1 or DG granule neurons by expressing a previously validated chained, triple MAGUK miR construct^60^ through biolistic transfection and stimulating Schaffer collaterals (Fig. 3a) or perforant pathways (Fig. 3d), respectively. Similar to a previous report^60^, we found that knocking down PSD-95, PSD-93, and SAP102 in CA1 pyramidal neurons led to reductions in both AMPAR- (Fig. 3b, g) and NMDAR-eEPSC amplitudes (Fig. 3c, h). When we repeated the experiment in DG granule neurons, we found that the resulting reductions in AMPAR- and NMDAR-mediated current amplitudes were nearly identical to the deficits in CA1 neurons following the same genetic manipulation (Fig. 3e–h). Our observation of very similar synaptic phenotypes in CA1 and DG granule neurons following knockdown of PSD-95, PSD-93, and SAP102 demonstrates that these MAGUK proteins do not play a unique role in DG granule neurons in general. Rather, these data demonstrate that βSAP97 plays a unique and vital synaptic regulatory role specifically in DG granule neurons in a manner that is distinct from other MAGUKs.

### Inhibition of βSAP97 function in the dentate gyrus disrupts contextual episodic memory

Microdeletion mutations resulting in reduced SAP97 expression have been linked to a 40-fold increase in the risk of developing schizophrenia^8,9^. Given that reduced βSAP97 expression results in dramatic augmentation of glutamatergic synapse strength in the DG granule neurons, we were interested in whether reduced βSAP97 expression within the dentate gyrus produces behavioral phenotypes associated with schizophrenia. A favored cognitive model of the abnormal behaviors and experiences characteristic of schizophrenia suggests that they may be linked to a disturbance in the effects of context^41,46–52^. More specifically, it has been suggested that individuals with schizophrenia exhibit a diminished ability to assess the relative importance of contextual cues in their environment, and that such a deficit may ultimately lead to the development of delusions, disorganization, hallucinations, and the loss of a sense of personal identity^40–45^. A prediction of this model of schizophrenia is that the normal effects of context on memory should be strongly reduced in patients with schizophrenia, as this type of information is believed to be poorly integrated into the episodic representation^69^. Thus, we were interested in whether reduced βSAP97 expression specifically in the dentate gyrus produces a deficit in contextual information processing in rodents.

**Fig. 4:**
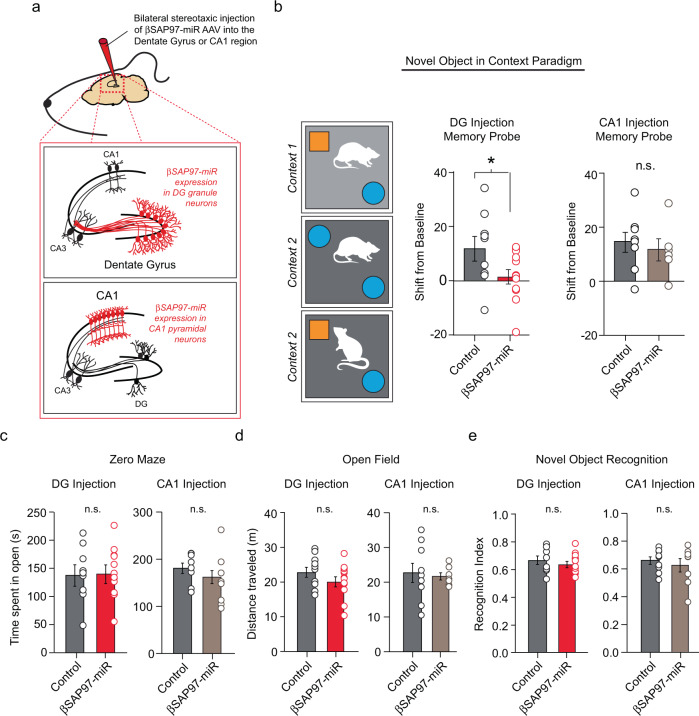
Knockdown of βSAP97 expression in the dentate gyrus disrupts contextual episodic memory. **a** Illustration of method used to inhibit βSAP97 function within the dentate gyrus or the CA1 region of the hippocampus. **b** (Left) Experimental setup of the Novel Object in Context behavioral procedure. (Center) Compared to control animals, βSAP97-miR expression in the dentate gyrus led to a significantly reduced exploration of the object that was novel in Context 2, expressed as a shift from baseline exploration for the same object in Context 1 (control *n* = 9, βSAP97 KD *n* = 12, *p* = 0.04, ANOVA). (Right) βSAP97-miR expression in the CA1 region led to no significant changes in exploration of the object that was novel in Context 2 (control *n* = 8, βSAP97 KD *n* = 6, *p* = 0.61, ANOVA). **c** Control and βSAP97 knockdown animals spent similar amounts of time in the open zones in a Zero Maze test for anxiety-like behavior following βSAP97-miR AAV injections in either the dentate gyrus (Left, control *n* = 10, βSAP97 KD *n* = 12, *p* = 0.91, two sample *T* test) or the CA1 region (Right, control *n* = 8, βSAP97 KD *n* = 8, *p* = 0.3, two sample *T* test). **d** Control and βSAP97 knockdown animals traveled similar distances in an Open Field test for exploratory behavior and general activity following βSAP97-miR AAV injections in either the dentate gyrus (Left, control *n* = 10, βSAP97 KD *n* = 12, *p* = 0.2, two sample *T* test) or the CA1 region (Right, control *n* = 9, βSAP97 KD *n* = 7, *p* = 0.76, two sample *T* test). **e** Recognition indices of control and βSAP97 knockdown animals were similar during a Novel Object Recognition task following βSAP97-miR AAV injections in either the dentate gyrus (Left, control *n* = 9, βSAP97 KD *n* = 12, *p* = 0.43, ANOVA) or the CA1 region (Right, control *n* = 9, βSAP97 KD *n* = 8, *p* = 0.54, ANOVA). *p < 0.05; n.s., not significant. All statistical tests performed were two-tailed. Source data are provided in the Source Data file.

To determine whether diminished βSAP97 expression specifically within the dentate gyrus affects the ability of animals to process contextual information, we performed bilateral stereotaxic injections of our AAV virus containing the βSAP97-miR expression construct into either the dentate gyrus or the hippocampal CA1 region of Sprague Dawley rats (Fig. 4a and Supplementary Fig. 4). Following viral transduction, we examined contextual episodic memory in these animals by assessing performance on a Novel Object in Context task^70–72^. In this task, rats were acclimated to a unique combination of objects in two different contexts, Context 1 and Context 2 (Fig. 4b). The animals were then placed in Context 2 containing the combination of objects from Context 1. One of these objects was previously unique to Context 1 and was novel in Context 2. Rats are capable of recognizing this novel, “out of context”, object as evidenced by increased exploration time (Fig. 4b). Our results revealed that compared to control animals, βSAP97-miR expression in the dentate gyrus led to a significantly reduced time spent exploring the out of context object in Context 2, expressed as a shift from baseline preference for the same object when presented in Context 1 (Fig. 4b; see “methods”). In other words, animals with compromised βSAP97 expression in the dentate gyrus did not appear to recognize this object as out of context. In marked contrast, expression of our βSAP97-miR in the CA1 region of the hippocampus produced no effect on rat performance on the Novel Object in Context task (Fig. 4b). To ensure that the decreased exploration times of the out of context object we observed with βSAP97-miR expression in the dentate gyrus were not secondary to a general avoidance of novel objects due to altered anxiety, we tested anxiety-like behavior using the Zero Maze test. Results showed no differences between groups in time spent in open zones, indicating no effect of dentate gyral βSAP97-miR expression on anxiety-related behavior (Fig. 4c). We also observed no effect of dentate gyral βSAP97-miR expression on locomotion as indicated by distance traveled in an open field (Fig. 4d). Furthermore, the inability of animals to identify the out of context object following βSAP97-miR expression in the dentate gyrus was not due to the inability of these animals to identify novel objects as evidenced by similar performance to controls on a standard perirhinal cortex-dependent Novel Object Recognition task (Fig. 4e). βSAP97-miR expression in the CA1 region of the hippocampus also did not affect performance on the Zero Maze, Open Field or Novel Object Recognition tasks (Fig. 4c–e). Thus, we conclude that compromised βSAP97 expression specifically within the dentate gyrus is sufficient to produce a substantial deficit in contextual information processing in rats. These data also demonstrate that disruption of βSAP97 function specifically within the dentate gyrus results in behavioral consequences that are related to cognitive impairments observed in individuals with schizophrenia.

### Schizophrenia-related mutations in βSAP97 release GluA1-containing AMPARs into perforant pathway synapses

**Fig. 5:**
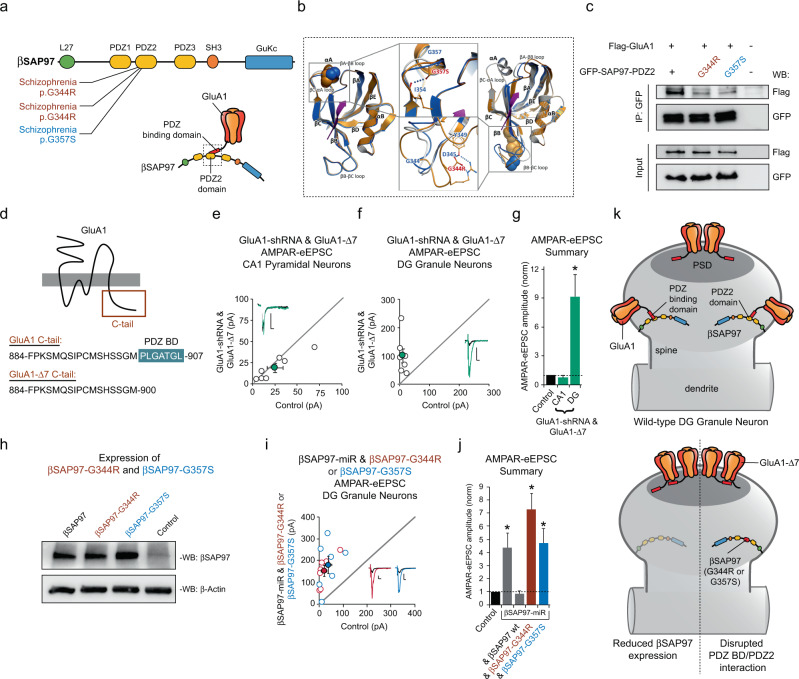
Clustered schizophrenia-related missense mutations in βSAP97’s PDZ2 domain release GluA1-containing AMPARs into perforant pathway synapses. **a** Illustration of βSAP97’s protein domain structure and location of schizophrenia-related missense mutations identified in SAP97 (Top). Illustration of interaction between GluA1’s PDZ-binding domain and βSAP97’s PDZ2 domain (Bottom, dashed box). **b** Protein structural modeling of schizophrenia-related mutations in βSAP97’s PDZ2 domain predict disruption of PDZ2’s interaction with GluA1’s PDZ-binding domain (see Supplementary Fig. 5 for more details). **c** Co-immunoprecipitation of FLAG-GluA1 with GFP-SAP97-PDZ2, GFP-SAP97-PDZ2(G344R), or GFP-SAP97-PDZ2(G357S) in HEK293T cells. See methods for details. Quantification for n = 3 independent experiments provided in Supplementary Fig. 6a. **d** Topology of the AMPAR subunit GluA1 and C-terminal amino acid sequences for GluA1 and GluA1-Δ7. GluA1’s PDZ-binding domain (PDZ BD) is highlighted in blue. For panels (**e**), (**f**), and (**i**), open circles in the scatter plots represent single pairs of control and transfected neurons, filled circles represent the mean amplitudes (±SEM), insets show representative current traces from control (black) and transfected (in color) neurons with stimulation artifacts removed. Scale bars: 20 ms, 20 pA. **e** Molecular replacement by co-expression of the GluA1-shRNA and GluA1-Δ7 has no effect on AMPAR-eEPSCs in CA1 pyramidal neurons (*n* = 7 pairs, *p* = 0.16, paired *T* test). **f** Molecular replacement by co-expression of GluA1-shRNA and GluA1-Δ7 significantly increases AMPAR-eEPSC amplitude in DG granule neurons (*n* = 7 pairs, *p* = 0.016, paired *T* test). **g** Summary bar graph for (**e**) and (**f**) shows the average AMPAR-eEPSC amplitudes (±SEM) of CA1 pyramidal neurons and DG granule neurons with co-expression of GluA1-shRNA and GluA1-Δ7 normalized to their respective control neurons. **h** Representative western blot analysis showing that βSAP97-G344R and βSAP97-G357S exhibit levels of expression in HEK293 cells similar to that of wild-type βSAP97 (*n* = 2 independent experiments). **i** Molecular replacement of βSAP97 with βSAP97-G344R and βSAP97-G357S in DG granule neurons significantly increases AMPAR-eEPSC amplitude (βSAP97-G344R, *n* = 7 pairs, *p* = 0.0004, paired *T* test; βSAP97-G357S, *n* = 7 pairs, *p* = 0.01, paired *T* test). **j** Summary bar graph shows the average AMPAR-eEPSC amplitudes (±SEM) of the conditions compared in panel (**i**). *p < 0.05. **k** Model of βSAP97-mediated regulation of glutamatergic synaptic function. GluA1-containing AMPARs on the surface of neurons are held perisynaptically by an interaction between βSAP97 and GluA1’s PDZ-binding domain. Reducing βSAP97 expression or inhibiting GluA1’s ability to bind to βSAP97’s PDZ2 domain results in local rearrangement of AMPA receptor organization in spines whereby AMPA receptors normally held by βSAP97 perisynaptically are released and then grabbed by PSD-95, PSD-93, and SAP102 in the PSD. All statistical tests performed were two-sided. Source data are provided in the Source Data file.

Missense mutations in SAP97 have been identified in individuals with schizophrenia and are clustered in SAP97’s PDZ2 domain^12,14^ (Fig. 5a). Two unrelated individuals with schizophrenia have the same missense mutation in SAP97, SAP97-G344R^12^ (Fig. 5a). An additional individual with schizophrenia was also identified harboring the de novo mutation SAP97-G357S^14^ (Fig. 5a).

SAP97 binds directly to AMPARs through an interaction between GluA1’s seven amino acid C-terminal PDZ-binding domain and SAP97’s PDZ2 domain^16–20^ (Fig. 5a). This is in contrast to PSD-95, PSD-93, and SAP102 which associate with AMPAR subunits through an intermediate interaction with transmembrane AMPAR regulatory proteins (TARPs)^73^. The structure of SAP97’s PDZ2 domain bound to GluA1’s C-terminal PDZ-binding domain has been solved^74^. Using this structure, our protein structural modeling predicted that the schizophrenia-related missense mutations identified in SAP97’s PDZ2 domain will inhibit SAP97’s ability to associate with GluA1’s PDZ-binding domain (Fig. 5b and Supplementary Fig. 5). Our modeling was then validated using co-immunoprecipitation of GluA1 with SAP97’s PDZ2 domain in HEK293T cells. As predicted, we found that schizophrenia-related missense mutations in SAP97’s PDZ2 domain significantly inhibit binding to GluA1 (Fig. 5c and Supplementary Fig. 6a).

Based on our results demonstrating increased AMPAR-eEPSC amplitude following knockdown of βSAP97 in DG granule neurons, we postulated that this increase in synaptic AMPAR expression was a result of reduced interaction between βSAP97’s PDZ2 domain and GluA1’s PDZ-binding domain. However, previous studies in CA1 pyramidal neurons have led groups to conclude that GluA1’s PDZ-binding domain is dispensable for glutamatergic neurotransmission^18,21^. If disruption of direct interaction between GluA1 and βSAP97 is responsible for the increase in AMPAR-eEPSC amplitude that we observe following knockdown of βSAP97 expression in DG granule neurons, then removing GluA1’s PDZ-binding domain and preventing its ability to bind to βSAP97 should also increase AMPAR-eEPSC amplitude. To test this idea, we employed a molecular replacement approach by biolistically co-transfecting a previously validated GluA1-shRNA construct^75^ and a form of shRNA-resistant GluA1 lacking the C-terminal PDZ-binding domain (GluA1-Δ7) (Fig. 5d) in both CA1 and DG granule neurons. Consistent with previous reports^18,21^, we found that molecular replacement of GluA1 with GluA1-Δ7 in CA1 pyramidal neurons has no effect on AMPAR-eEPSC amplitude (Fig. 5e, g). In marked contrast, we found that molecular replacement of GluA1 with GluA1-Δ7 in DG granule neurons phenocopied knockdown of βSAP97, producing a dramatic increase in AMPAR-eEPSC amplitude (Fig. 5f, g). We also assessed whether replacement of GluA1 with GluA1-Δ7 occludes a further increase in AMPAR-eEPSC amplitude produced by βSAP97 knockdown in DG granule neurons. We found that further augmentation was indeed occluded (Supplementary Fig. 6b), suggesting that the augmented synaptic strength following GluA1 replacement with GluA1-Δ7 and following βSAP97 knockdown in DG granule neurons results from disruption of the same molecular mechanism. Taken together, these data demonstrate that a direct interaction between GluA1’s PDZ-binding domain and βSAP97 mediates βSAP97’s ability to inhibit synaptic AMPAR expression in DG granule neurons. We hypothesized from these data that reducing βSAP97 expression releases AMPARs from perisynaptic regions which are in turn captured by traditional MAGUKs at the synapse. If such was the case, knocking down these traditional MAGUK proteins at the synapse would prevent the βSAP97 knockdown-mediated augmentation of DG granule neuron synapse function. To test this, we co-expressed the triple MAGUK miR construct with our βSAP97-miR in DG granule neurons, and, consistent with our hypothesis, we found that βSAP97 knockdown does not augment AMPAR-eEPSC amplitude in DG granule neurons on the PSD-95, PSD-93, and SAP102 knockdown background (Supplementary Fig. 6c).

Together, our data suggest that schizophrenia-related missense mutations that inhibit the ability of βSAP97’s PDZ2 domain to interact with GluA1 (Fig. 5c) should produce a potentially pathological release of GluA1-containing AMPARs into the glutamatergic synapses of DG granule neurons. Before directly testing whether schizophrenia-related missense mutations in βSAP97’s PDZ2 domain influence synaptic AMPAR function, we first examined the expression of wild-type βSAP97, βSAP97-G344R, and βSAP97-G357S. We observed levels of βSAP97-G344R and βSAP97-G357S expression that were comparable to wild-type βSAP97 (Fig. 5h). Additionally, we observed targeting of βSAP97-G344R and βSAP97-G357S to dendritic spines in DG granule neurons like that observed with wild-type βSAP97 (Supplementary Fig. 6d, 2d). We then examined AMPAR-eEPSC amplitude in DG granule neurons where endogenous βSAP97 was molecularly replaced with βSAP97-G344R or βSAP97-G357S (Fig. 5a, i, j). In contrast to molecular replacement with wild-type βSAP97 (Figs. 1d, 5j), we found that both schizophrenia-related mutant forms of βSAP97 produced dramatic increases in synaptic AMPAR-eEPSC amplitude in DG granule neurons like that seen with knockdown of βSAP97 and molecular replacement of GluA1 with GluA1-Δ7 (Fig. 5i, j). Molecularly replacing βSAP97 with βSAP97-G344R, which produced the largest increase in AMPAR-eEPSC amplitude of the two missense mutations, was found to have no effect on dendritic AMPAR surface current (Supplementary Fig. 6e). As with reduced βSAP97 expression, such data demonstrate that the AMPAR-eEPSC augmentation produced by these schizophrenia-related missense mutations results from rearrangement of AMPARs on the dendritic surface of DG granule neurons. Finally, we performed quantal analysis on our βSAP97 knockdown AMPAR-eEPSC data and on our AMPAR-eEPSC data from molecular replacements with the schizophrenia-related mutants to determine whether the augmented AMPAR-eEPSC amplitude in these conditions is caused by an increase in quantal size or quantal content^54,55,57,60,76,77^. We found that the augmentations in all three conditions were caused by an increase in quantal size rather than quantal content (Supplementary Fig. 6f). Thus, the increases in AMPAR-eEPSC amplitude we observe are due to increased AMPAR expression at existing functional glutamatergic synapses rather than an increase in glutamatergic synapse number.

Altogether, our data demonstrate that both reduced βSAP97 expression and schizophrenia-related missense mutations in βSAP97’s PDZ2 domain compromise βSAP97’s interaction with GluA1-containing AMPARs and, as a result, cause rearrangements of AMPARs present on the dendritic surface of DG granule neurons that lead to increased AMPAR expression in the PSDs of existing functional glutamatergic synapses. We observe robust localization of βSAP97 in the spines of DG granule neurons that is consistent with previous work demonstrating a perisynaptic localization of βSAP97^61^. Therefore, we believe the most likely explanation for our findings is that βSAP97 maintains perisynaptic pools of AMPARs in DG granule neurons (Fig. 5k), and that inhibiting βSAP97’s interaction with GluA1 by way of reduced βSAP97 expression or missense mutations in βSAP97’s PDZ2 domain causes AMPARs to translocate from these perisynaptic regions to the PSD resulting in aberrant increases in perforant pathway synapse strength (Fig. 5k). Such elevations in the strength of perforant pathway synapses likely disrupt dentate gyral information processing and may contribute to the development of behavioral phenotypes associated with schizophrenia.

## Discussion

While the synaptic roles of most MAGUK proteins are well understood (i.e. PSD-95, PSD-93, and SAP102), the role of SAP97 in regulating glutamatergic neurotransmission has remained unclear. In previous studies, overexpression of recombinant SAP97 in neurons has led to mixed and inconclusive results^22–27^. It is difficult to derive meaningful conclusions from such experiments given inconsistencies in the SAP97 splice variants used across studies. Furthermore, overexpressing recombinant proteins in neurons that do not utilize the protein endogenously may lead to aberrant protein function. Inhibiting SAP97 expression has also been shown to produce a variety of seemingly contradictory glutamatergic synapse phenotypes^22,25,28^. Such discrepancies have likely arisen at least in part because of the different neuronal preparations used. In the case of dissociated neuron cultures, neurons lose specificity in their synaptic connections and instead form synapses with neighboring neurons regardless of the original circuitry. In such artificial contexts, neurons may develop synapses utilizing proteins they otherwise would not. Critical controls for SAP97 RNAi usage, such as rescue experiments, are also missing in previous studies making it difficult to rule out off-target effects. Thus, previous literature has led to considerable confusion regarding SAP97’s role in synaptic regulation and, based on the absence of a synaptic phenotype in CA1 pyramidal neurons following knock out of SAP97^28^, groups have generally concluded that SAP97 has no role in the regulation of glutamatergic synapse function^15^.

Driven by the implication of dentate gyral and SAP97 dysfunction in schizophrenia, the present study visually identified the perforant pathway-DG granule neuron synapse as where SAP97 might play an important synaptic role due to the robust endogenous βSAP97 expression in the dendrites of DG granule neurons. We determined that inhibiting βSAP97 function specifically in DG granule neurons leads to a dramatic increase in AMPAR-mediated currents, signaling an increase in synaptic AMPAR expression. In contrast, we observed no effects on synaptic transmission following knockdown of βSAP97 in CA1 pyramidal neurons, as predicted from the lack of dendritic expression of βSAP97 in CA1 neurons. Taken together, our data demonstrate that βSAP97 plays a critical cell-type-specific regulatory role at perforant pathway-DG granule neuron synapses. Thus, we have identified a specific set of synapses where βSAP97 is essential for regulating glutamatergic neurotransmission. In contrast to a previous study performed in dissociated hippocampal neurons^25^, we find that βSAP97 does not play a significant role in governing synaptic NMDAR function. The lack of an effect we observe on NMDAR function is consistent with previous biochemical evidence suggesting that SAP97 does not interact with NMDARs^16,78^. One possible explanation for this discrepancy is that the formation of glutamatergic synapses between hippocampal neurons outside of their native circuitry confers unique properties to βSAP97. Intriguingly, we also observe a laminar pattern of βSAP97 immunolabeling within the molecular layer of the dentate gyrus that is consistent with higher levels of βSAP97 expression in the synapses of the lateral perforant pathway relative to the medial perforant pathway. Going forward, it will be interesting to determine if βSAP97 inhibition of AMPAR-mediated synaptic transmission is greater at lateral perforant pathway synapses compared to those of the medial perforant pathway.

Having identified the synapses where SAP97 plays a critical role, we began characterizing the mechanism by which βSAP97 regulates synaptic AMPAR expression. We found through IV rectification analysis that βSAP97 appears not to govern synaptic AMPAR subunit composition. GABAergic neurotransmission and intrinsic excitability were also unaffected by βSAP97 knockdown in DG granule neurons. Furthermore, we determined through exogenous glutamate application that while the synaptic AMPAR function increased following βSAP97 knockdown, AMPAR expression on the surface of DG granule neuron dendrites did not change. This demonstrates that the increase in synaptic AMPAR function we observe is not due to the exocytosis of intracellular AMPARs but rather a reorganization of AMPARs on the dendritic surface (Fig. 5k). Additionally, we found that knocking down MAGUKs PSD-95, PSD-93, and SAP102 decreased synaptic transmission similarly in CA1 pyramidal neurons and DG granule neurons. This observation ruled out the possibility that the dramatic increase in synaptic AMPAR expression following βSAP97 knockdown in DG granule neurons was due to all MAGUK proteins playing a fundamentally different role in the dentate gyrus. Thus, our data demonstrate that βSAP97 is distinct from the other MAGUKs in its function.

In humans, microdeletion mutations involving the *DLG1* gene are believed to lower βSAP97 expression and contribute to a greater than 40-fold increase in the risk of developing schizophrenia^8,9^. The development of schizophrenia-related disorders has also been linked to disruption of dentate gyral information processing^1,30–38^. Given that reduction of βSAP97 expression resulted in dramatic augmentation of glutamatergic synapse strength in DG granule neurons, we were interested in whether reducing βSAP97 expression specifically within the dentate gyrus is sufficient to produce behavioral phenotypes associated with schizophrenia. A well-established model of schizophrenia suggests that deficits in processing contextual information during episodic memory formation may underlie core phenotypes associated with schizophrenia including delusions, disorganization, hallucinations, and the loss of a sense of personal identity^40–45^. Here, we investigated contextual episodic memory formation in rats following the expression of our βSAP97-miR in the dentate gyrus or the CA1 region of the hippocampus. We found that animals with βSAP97-miR expression in the dentate gyrus exhibited substantial impairment in contextual information processing during episodic memory formation. In marked contrast, βSAP97-miR expression in the CA1 region of the hippocampus produced no effect on contextual information processing. Thus, contextual information processing is only compromised by our βSAP97-miR in the brain region where we observe dendritic expression of βSAP97 and where glutamatergic synapse function is augmented when βSAP97 expression is knocked down. Together, our results establish that disruption of βSAP97 function specifically within the dentate gyrus is sufficient to produce behavioral deficits consistent with those observed in individuals with schizophrenia. Given that we see no change in GABAergic neurotransmission or intrinsic excitability in DG granule neurons following knockdown of βSAP97 expression, we believe that the augmented glutamatergic synapse strength we observe in these neurons is likely responsible for the deficit in contextual information processing in these animals. However, we do acknowledge that we cannot definitively exclude the relevance of some yet-to-be-discovered role of βSAP97 in regulating DG neuron function.

Traditional MAGUK proteins (PSD-95, PSD-93, and SAP102) interact with AMPARs through TARP proteins, which contain PDZ-binding motifs^73^. βSAP97 differs from these MAGUKs in that its PDZ2 domain can directly interact with the C-terminal PDZ-binding domain of the AMPAR subunit GluA1^16–20^. In humans, schizophrenia-related missense mutations in SAP97 are clustered in SAP97’s PDZ2 domain and here we show that these mutations inhibit GluA1 binding to SAP97’s PDZ2 domain. These data suggested that inhibition of SAP97’s interaction with GluA1 may be responsible for the increased synaptic AMPAR function we observe in DG granule neurons, which may, in turn, contribute to the development of schizophrenia in humans. In contrast to traditional MAGUK proteins, βSAP97 possesses an N-terminal L27 domain that causes βSAP97 to localize to perisynaptic regions in spines outside of the PSD^61,79^. Based on this previous work and our own βSAP97 localization data, we hypothesized that endogenous βSAP97 may sequester GluA1-containing AMPARs perisynaptically in DG granule neurons through a direct interaction between βSAP97 and GluA1 subunits. Reduced βSAP97 expression or schizophrenia-related missense mutations in βSAP97’s PDZ2 domain may disrupt the direct interaction between βSAP97 and GluA1’s C-terminal PDZ-binding domain causing AMPARs to be released into the PSD. If this is the case, removing GluA1’s PDZ-binding domain in DG granule neurons should result in elevated synaptic AMPAR function like that produced by βSAP97 knockdown. Removal of GluA1’s PDZ-binding domain (GluA1-Δ7) was previously examined in CA1 pyramidal neurons and produced no effect on glutamatergic neurotransmission, suggesting that in these neurons a GluA1-βSAP97 interaction likely does not play a regulatory role^18^. In fact, the relevance of GluA1’s entire C-tail in the regulation of glutamatergic neurotransmission was recently called into question^21^. In agreement with these studies, we found that molecularly replacing endogenous GluA1 with GluA1-Δ7 in CA1 pyramidal neurons produced no effect on baseline glutamatergic neurotransmission. However, when repeating the experiment in DG granule neurons, we found a dramatic increase in AMPAR-eEPSC amplitude that phenocopies what is observed with knocking down βSAP97. We also found that GluA1-Δ7 molecular replacement in DG granule neurons prevents further augmentation of synaptic strength by our βSAP97-miR. Together, such data demonstrate that disruption of βSAP97’s interaction with GluA1’s C-terminal PDZ-binding domain produces the dramatic increases in AMPAR-eEPSC amplitude that we observe in DG granule neurons (Fig. 5k).

Our protein structural modeling and co-immunoprecipitation data demonstrate that schizophrenia-related mutations clustered in βSAP97’s PDZ2 domain inhibit GluA1’s ability to bind to this domain. Such data led us to predict that these mutant forms of βSAP97 would produce pathological augmentation of synaptic AMPAR function in DG granule neurons. Indeed, we find that molecular replacement of βSAP97 with these schizophrenia-related mutant forms of βSAP97 produce dramatic augmentation of AMPAR-eEPSC amplitude in DG granule neurons like that seen with βSAP97 knockdown and molecular replacement of GluA1 with GluA1-Δ7. Quantal analysis performed on these data demonstrate that the increase in AMPAR-eEPSC amplitude that we observe with these mutations is due to increased AMPAR expression at existing functional glutamatergic synapses. This finding is in line with previous work showing that recombinant βSAP97 accumulates in dendritic spines as they structurally mature^80^. We also show that these mutations do not impact βSAP97 expression, targeting of βSAP97 to spines, or dendritic AMPAR surface current. Altogether, our data suggest that βSAP97 binds to the C-terminal PDZ domain of GluA1-containing AMPARs and, through this interaction, maintains a perisynaptic surface pool AMPARs within the spines of DG granule neurons (Fig. 5k). This mechanism is consistent with that proposed by a previous study performed utilizing recombinant βSAP97 in neurons^61^. Furthermore, our data suggest that when the interaction between βSAP97 and GluA1 is disrupted, either by reducing βSAP97 expression or schizophrenia-related missense mutations in βSAP97’s PDZ2 domain, AMPARs are released from perisynaptic sites into the PSD causing a pathological increase in synaptic strength in DG granule neurons (Fig. 5k) that likely disrupts information processing in the dentate gyrus. Going forward, it will be important to determine why DG granule neurons uniquely employ βSAP97 to sequester AMPARs outside of the PSD.

In conclusion, our study identifies a cell-type-specific synaptic regulatory mechanism in the dentate gyrus that, when disrupted, impairs contextual information processing in rats. As a result, restoring proper βSAP97 function and/or reducing AMPAR-mediated neurotransmission within the dentate gyrus should be considered when designing potential therapeutic strategies for individuals harboring pathological mutations in the *DLG1* gene. In future studies, it will also be important to determine whether similar synaptic pathology in the dentate gyrus is produced by schizophrenia-related mutations in other genes. Information derived from such studies will be instrumental in establishing how broadly applicable these therapeutic strategies might be in the treatment of this psychiatric disorder.

## Methods

### Experimental constructs

βSAP97-miR target sequence 5′-TCTACTGGAGGGCTAAGGCCG-3′ was embedded into an emerald GFP (emGFP) sequence in a pFUGW expression vector to make a pFUGW-βSAP97-miR construct. For the SAP97 rescue experiment, cDNA for RNAi-resistant human βSAP97 with inserts I1B, I3, and I5 was obtained from GE Dharmacon (CloneId: 9053182) and cloned into NheI and XmaI sites of a pCAGGS-IRES-mCherry expression vector. Schizophrenia-related missense mutants βSAP97-G344R and βSAP97-G357S were made from this pCAGGS-βSAP97-IRES-mCherry construct using overlap-extension PCR followed by In-Fusion cloning (Clontech). mCherry-tagged βSAP97 constructs (wild-type, G344R and G357S) were made by deleting the IRES element and fusing the mCherry onto the C-terminus of βSAP97 in the pCAGGS-βSAP97-IRES-mCherry and mutant constructs. The chained, triple MAGUK miR construct was previously validated^60^ and was generously provided by Dr. Roger Nicoll. Rat GluA1 (*flip*-type) construct pCAGGS-GluA1-IRES-GFP, pCAGGS-FLAG-GluA1, and the FHUGW-GluA1shRNA construct (target sequence 5’ - GGAATCCGAAAGATTGGTT – 3’) were generously provided by Dr. Katherine Roche^75^. An RNAi-resistant form of GluA1 was generated by introducing five silent mutations within the shRNA target sequence via overlap-extension PCR followed by In-Fusion cloning (Clontech). shRNA-resistant GluA1 missing the C-terminal PDZ-binding domain (GluA1-Δ7), specifically missing the last seven amino acid residues PLGATGL, was generated also using the overlap-extension PCR and In-Fusion methods. All plasmids were confirmed by DNA sequencing. Oligonucleotide sequences used to generate experimental constructs are provided in Supplementary Table 1.

### Immunohistochemistry

Experiments were performed in accordance with NIH Guidelines for the Care and Use of Laboratory Animals, and all procedures were approved by the Institutional Animal Care and Use Committee of the University of Southern California. Postnatal day 15 (P15) Sprague Dawley rats of both sexes were transcardially perfused with 11 ml of cold PBS and 25 ml of cold 4% PFA in PBS at a flow rate of 3 ml/min. The hippocampi were immediately dissected and were post-fixed overnight at 4 °C in 4% PFA. After 3 brief washes in PBS, the hippocampi were sliced using a vibratome at 100μm thickness. Slices were placed into 24-well culture plates containing PBS and stained within the wells. Slices were blocked in PBST (PBS + 0.25% TritonX-100) with 10% Goat Serum for 1 h at room temperature, rinsed in PBST, and incubated with primary antibody diluted in PBST overnight at 4 °C. Then the slices were thoroughly washed in PBST and stained with secondary antibody diluted in PBST for 2 h at room temperature. Slices were then mounted onto slides, dried for 15 min, and mounted with either Fluoromount-G (SouthernBiotech, Cat#0100-01) or Fluoroshield with DAPI (Sigma Aldrich, Cat#F6057). For the immunizing peptide block experiment, a blocking peptide matching the epitope sequence of the rabbit anti-SAP97 antibody EEYRSKLSQTEDRQLRSS was synthesized (>98% purity). Diluted SAP97 primary antibody was prepared as normal, the blocking peptide was added to the antibody at a 10:1 ratio, and the mixture was incubated overnight at 4 °C. This peptide-blocked antibody was used following the same staining protocol as detailed above. Antibodies used are as follows: rabbit anti-SAP97 (1:1000, Invitrogen Cat#PA1-044, RRID: AB_2092021), mouse anti-MAP2 (1:1000, Sigma Aldrich Cat#4403), rabbit IgG polyclonal isotype control antibody (1:1000, Abcam Cat#171870), rabbit anti-SAP97 (100ug, custom made, YenZym Antibodies, epitope sequence: DQSEQETSDADQ), goat anti-rabbit Alexa Fluor 555 (1:1000, Invitrogen Cat#A32732, RRID: AB_2633281), goat anti-mouse Alexa Fluor 488 (1:1000, Invitrogen Cat#A-11001, RRID: AB_2534069). Slides were imaged with a Keyence All-in-One Fluorescence Microscope BZ-X800 with a 4x objective for whole hippocampal slice imaging and with Zeiss 880 Confocal Microscope with 10x and 40x water-immersion objectives.

### AAV production

Our emGFP-βSAP97-miR expression construct was subcloned into AAV-bGH(+) and packaged into an adeno-associated virus (AAV2; Vector Biolabs) under the control of a UbC promoter to create the βSAP97 miR AAV (titer = 1.0e13 GC/ml). A scrambled miR, emGFP-expressing AAV2 downstream of a UbC promoter (titer = 2.1e13 GC/ml) was used as a control (Vector Biolabs).

### Immunoblotting and Co-immunoprecipitation

For knockdown experiments in primary rat hippocampal dissociated neurons, neurons were prepared from E18.5 Sprague Dawley rats (Charles River Laboratories, Wilmington, MA, USA) of both sexes and transduced with either βSAP97-miR AAV or scrambled-miR AAV at DIV1. Lysates were prepared at DIV14 in RIPA buffer containing protease inhibitor mix (ThermoFisher, Halt Protease Inhibitor Cocktail). Proteins were resolved by SDS-PAGE. Following the transfer, membranes were cut and analyzed by western blot with antibodies against SAP97 (1:1000, Invitrogen, Cat#PA1-741, RRID: AB_2092020), PSD-95 (1:1000, Millipore, Cat#637258), PSD-93 (1:1000, Millipore, Cat#618436), SAP102 (1:1000, Biolegend, Cat#832004), and β-actin (1:1000, Cell Signaling Technology, Cat#4970S). Goat anti-rabbit, HRP-linked secondary antibody (1:10000, Cell Signaling Technology, Cat#7074S) or horse anti-mouse, HRP-linked secondary antibody (1:10000, Cell Signaling Technology, Cat#7076S) were used for all immunoblotting experiments described. For validating βSAP97 rescue by the miR-resistant βSAP97 construct, HEK293 cells (ATCC, Cat#CRL-1573) cultured in DMEM supplemented with 10% FBS and 1% penicillin-streptomycin and maintained at 37 °C and 5% CO2 were transfected using Lipofectamine 2000 (Invitrogen, Cat#11668027) with the following constructs: GFP & βSAP97, βSAP97-miR & βSAP97, or βSAP97-miR & βSAP97 miR-resistant. Cells were harvested 72 h post-transfection. Lysates were prepared in RIPA buffer containing protease inhibitor mix, and proteins were resolved by SDS-PAGE. Following the transfer, membranes were cut and analyzed by western blot with antibodies against SAP97 (1:500, Neuromab, Cat#75-030) and β-actin (1:1000, Cell Signaling Technology, Cat#4970S). For schizophrenia mutant expression experiments, HEK293 cells were transfected with cDNAs of βSAP97, βSAP97-G344R, or βSAP97-G357S using Lipofectamine 2000 and harvested 72 h post-transfection. Lysates were prepared in RIPA buffer containing protease inhibitor mix, and proteins were resolved by SDS-PAGE. Following the transfer, membranes were cut and analyzed by western blot with the same SAP97 and β-actin antibodies and concentrations as the experiments described above.

For co-immunoprecipitation experiments, HEK293T cells (ATCC, Cat#CRL-3216) were co-transfected with FLAG-GluA1 and wild-type GFP-SAP97-PDZ2, GFP-SAP97-PDZ2(G344R), or GFP-SAP97-PDZ2(G357S) using Lipofectamine 2000. 24 h following transfection, cells were washed and lysed (lysis buffer: 25 mM Tris-HCl pH 7.4, 150 mM NaCl, 1 mM EDTA, 1% NP-40, 5% glycerol). Lysates were rocked for 30 min at 4 °C and centrifuged. Supernatants were collected and incubated with anti-GFP antibody (mouse, Neuromab, Cat#75-131) overnight at 4 °C. Protein G Dynabeads (ThermoFisher, Cat#10007D) were added to the lysate/antibody mixture and incubated at 4 °C for 4 h. Beads were washed and eluted in the Dynabead elution buffer at room temperature to ensure that FLAG-GluA1 was detectable at the expected monomeric molecular weight. Whole-cell lysates used as inputs were boiled to improve resolution and band signal for both FLAG-GluA1 and GFP-SAP97-PDZ2. Proteins were resolved by SDS-PAGE. Following the transfer, membranes were cut and analyzed by western blot with antibodies against FLAG (1:2000, Sigma Aldrich, Cat#8592) and GFP (rabbit, 1:1000, Invitrogen, Cat#A-11122). Blots were quantified using Fiji. To compare the interactions between FLAG-GluA1 and WT, G344R, or G357S GFP-SAP97-PDZ2, bands representing immunoprecipitated GFP-SAP97-PDZ2, co-immunoprecipitated FLAG-GluA1, and input FLAG-GluA1 were measured. The fraction of input FLAG-GluA1 that was co-immunoprecipitated was calculated by dividing the co-immunoprecipitated FLAG-GluA1 by input FLAG-GluA1. This number was normalized to immunoprecipitated GFP-SAP97-PDZ WT, G344R, or G357S, resulting in the final numbers used to compare WT, G344R, and G357S GFP-SAP97-PDZ2, respectively. All uncropped blots with molecular weight ladders are provided in the Source Data.

### Real-time polymerase chain reaction

Rat hippocampal neurons were prepared from E18.5 Sprague Dawley rats (Charles River Laboratories, Wilmington, MA, USA) of both sexes and transduced with either βSAP97-miR AAV or scrambled miR AAV at DIV1. At DIV14 cells were suspended in a lysis buffer containing 1% beta-mercaptoethanol and disrupted using QIAshredder homogenizers (Qiagen, Cat#79654). Total RNA was purified and isolated with the RNeasy Micro kit (Qiagen, Cat#74004) following the manufacturer’s instructions. Total RNA content was quantified using the Nanodrop One spectrophotometer (ThermoFisher, Cat#ND-ONE-W) and the Quantitect Reverse Transcription kit was employed to synthesize complimentary DNA from 500 ng of total RNA (Qiagen, Cat#205311). Real-time polymerase chain reaction (RT-PCR) was run on a QuantStudio 5 RT-PCR system (Applied Biosystems, Cat#A28140) using the Taqman Fast Advanced Master Mix (Applied Biosystems, Cat#4444557) and Taqman Gene Expression Assay Mix for GAPDH (Assay ID Rn01775763_g1), αSAP97 (Assay ID ART2CT4) and βSAP97 (Assay ID Rn01439452_m1). CT values were obtained from the QuantStudio 5 Design & Analysis software and converted to fold changes using the Delta-Delta CT method.

### Electrophysiology

400 μm rat organotypic entorhino-hippocampal slice cultures were prepared from both male and female P6 to P8 Sprague Dawley rats as previously described^81–83^. Hippocampi with accompanying entorhinal cortices were removed from 6 to 10 rats at a time, and 400 μm transverse sections were made using a MX-TS tissue slicer (Siskiyou). Slices from these 6-10 rats were mixed together and then mounted on individual squares of Biopore Membrane filter roll (Millipore) and placed on Millicell Cell Culture inserts (Millipore) in 35 mm dishes containing 1 ml of culture media (MEM + HEPES (Gibco Cat#12360-038), horse serum 25%, HBSS (25%) and L-glutamine (1 mM). Media was exchanged every other day. Slices with large portions of entorhinal cortex were visually identified subsequent to slicing. These slices were selected and plated for use in our experiments, and the presence of entorhinal cortex was again confirmed when selecting slices appropriate for data acquisition. Culture media was exchanged every other day. Sparse biolistic transfections were performed on DIV1 as described in detail previously^23^. 50ug total of mixed plasmid DNA was coated on 1μm-diameter gold particles in 0.5 mM spermidine, precipitated with 0.1 mM CaCl_2_, and washed four times in pure ethanol. These DNA-coated gold particles were then coated onto PVC tubing, dried using ultra-pure N_2_ gas, and stored at 4 °C in desiccant. Before use, the gold particles were brought up to room temperature and then delivered to slice cultures via a Helios Gene Gun (BioRad). For biolistically transfecting more than one construct, equal amounts of plasmid DNA for each construct was used. Construct expression was confirmed by GFP or mCherry epifluorescence. Electrophysiological recordings were performed on DIV7 slices. During recordings, slices were maintained in room-temperature artificial cerebrospinal fluid (aCSF) external solution containing (in mM): 119 NaCl, 2.5 KCl, 1 NaH_2_PO_4_, 26.2 NaHCO_3,_ 11 glucose, 4 CaCl_2_, and 4 MgSO_4_. 5 μM 2-chloroadenosine and 0.1 mM picrotoxin were also added to the aCSF to dampen epileptiform activity and block GABA_A_ receptor activity, respectively. Osmolarity was adjusted to 310–315 mOsm. aCSF was saturated with 95% O_2_/5% CO_2_ throughout the recording. Borosilicate recording electrodes were filled with an internal, whole-cell recording solution containing (in mM): 135 CsMeSO_4_, 8 NaCl, 10 HEPES, 0.3 EGTA, 5 QX-314, 4 Mg-ATP, and 0.3 Na-GTP. The internal solution was adjusted to pH 7.3-7.4 and osmolarity of 290-295 mOsm.

Transfected DG granule neurons and CA1 pyramidal neurons were identified using epifluoresence microscopy. Dual whole-cell recordings of either neuronal subtype were made through simultaneous recordings from one transfected neuron and a neighboring non-transfected control neuron. Postsynaptic responses were evoked by stimulating with a monopolar glass electrode filled with aCSF placed in the middle of the molecular layer to stimulate both medial and lateral perforant pathway afferents for DG granule neuron recordings and at the stratum radiatum/stratum lacunosum-moleculare border or in the stratum oriens for CA1 pyramidal neuron recordings. The responses were acquired using a Multiclamp 700B amplifier (Molecular Devices), filtered at 2 kHz, and digitized at 10 kHz. AMPAR evoked EPSCS (eEPSCs) were measured at -70mV. NMDAR-eEPSCs were measured at +40 mV and were temporally isolated by measuring amplitudes 150 ms following the stimulus, at which point the AMPAR-eEPSC had completely decayed. Data analysis was performed using Igor Pro (Wavemetrics). In the scatter plots for simultaneous dual whole-cell recordings, each open circle represents one paired recording, and the closed circle represents the average of all paired recordings. The diagonal line is shown on the scatter plot to demonstrate that if the data point falls above the diagonal line, it indicates that the eEPSC is lower in the control neuron, and vice versa. No more than one paired recording was performed on any given entorhino-hippocampal slice. To elicit inhibitory synaptic responses, the glass monopolar stimulating pipette was placed in the molecular layer of the dentate gyrus. 100 uM D-APV and 10 uM NBQX were added to a picrotoxin-free external solution to isolate GABAR-eIPSCs.

For IV rectification analysis, 0.1 mM spermine was added to the internal solution described above for measurement of AMPA receptor-mediated current rectification. Rectification indices were calculated as the normalized glutamate-evoked current at +40 mV over −70 mV, respectively, in presence of 100 μM APV to block NMDAR-mediated EPSCs.

For measuring surface AMPAR currents, a picospritzer II (General Valve Co.) was used to puff-apply L-glutamate onto the dendrites of DG granule neurons in the molecular layer or onto the apical dendrites of CA1 pyramidal neurons during recording. Glutamate pulses of 10 ms were applied to patched neurons held at −70mV by a glass pipette. L-glutamate (25 mM) was applied to cells in a solution containing (in mM) NaCl 140, KCl 5, MgCl_2_ 1.4, CaCl_2_ 1, EGTA 5, and pH adjusted to 7.4. 100 uM APV was added to isolate AMPAR currents.

For current-clamp recordings, the intracellular solution contained (in mM) 130 KMeSO_4_, 10 KCl, 10 HEPES, 4 NaCl, 1 EGTA, 4 Mg-ATP, and 0.3 Na-GTP. 500 ms square current pulses were delivered to neurons held in current-clamp mode. For each recording, current pulse amplitude was increased from 10 to 110 pA in 10 pA increments. Rheobase values were defined as the minimum injected current required to elicit a single spike.

### βSAP97 localization imaging

DG granule neurons in organotypic entorhino-hippocampal slice cultures made from P6-P8 rats were biolistically co-transfected with mCherry-tagged βSAP97 constructs (wild-type, G344R, or G357S) and the GFP expressing pFUGW-βSAP97-miR construct ~18-20 h after plating. Slices were fixed in 4% PFA/4% Sucrose in PBS and washed 3x with PBS. Slices were further processed with an abbreviated SeeDB-based protocol for imaging^54,84,85^. Images were acquired at DIV7 via multiphoton confocal microscopy (SP8 LIGHTNING Confocal Microscope, Leica). Images were acquired using a 63x/1.4NA oil immersion objective. Imaris image analysis software (Oxford Instruments) was used to identify and visualize dendritic regions exhibiting the highest βSAP97 fluorescent intensity.

### Protein modeling

The high-resolution crystal structures of the SAP97 PDZ2 domain (PDB ID: 2AWX, 1.80 Å resolution; https://www.rcsb.org/structure/2AWX) and the SAP97 PDZ2 domain in complex with a GluA1 C-terminal peptide (PDB ID: 2G2L, 2.35 Å resolution) were used to predict the effect of mutations on binding GluA1’s PDZ-binding domain. Calculations were performed using ICM molecular modeling software (Molsoft LLC). Our modeling showed that mutation G357S results in the formation of a hydrogen bond between the hydroxyl group of S357 and carbonyl oxygen of I354. Substitution of flexible Glycine to Serine and formation of an additional hydrogen bond reduces the flexibility of the βC-αA loop. Moreover, this change may affect the conformational changes of βA-βB observed upon peptide binding, as βA-βB and βC-αA loops are located in close proximity to each other. The G344R mutation was predicted to impact the conformation of βB- βC loop, as G344 has torsion angles that are not compatible with other amino acid residues.

### Behavior—animals and surgery

For all behavior experiments, male Sprague Dawley rats (Envigo, Indianapolis, IN, USA) weighing 300–400 g (~P90) were individually housed in wire-hanging cages in a climate controlled (22–24 °C) environment with a 12:12 h light/dark cycle. Rats were given ad libitum access to water and standard rodent chow (LabDiet 5001, LabDiet, St. Louis, MO). Experiments were performed in accordance with NIH Guidelines for the Care and Use of Laboratory Animals, and all procedures were approved by the Institutional Animal Care and Use Committee of the University of Southern California.

For stereotaxic injection of AAVs for in vivo knockdown of SAP97 expression in the dorsal dentate gyrus (dDG) or in the dorsal CA1, rats were first anesthetized and sedated with a ketamine (90 mg/kg)/xylazine (2.8 mg/kg)/acepromazine (0.72 mg/kg) cocktail. Animals were then shaved and the surgical site was prepped with iodine and ethanol swabs before being placed in a stereotaxic apparatus for stereotaxic injections. βSAP97-miR AAV and scrambled-miR AAV were prepared by Vector Biolabs, as described above. AAVs were delivered bilaterally to either the dDG (AP: −3.12, ML: +/-1.20, DV:−3.9) or the dorsal CA1 (AP: −3.12, ML:  + /- 1.50, DV: −3.0) at an injection volume of 200 nl per hemisphere via pressure injections. Injections were administered using a microinfusion pump (Harvard Apparatus, Holliston, MA) connected to a 33-gauge microsyringe injector attached to a PE20 catheter and Hamilton syringe. The flow rate was calibrated and set to 5 μl/min. Injectors were left in place for 2 min to allow for complete infusion of the drug. Behavioral experimental procedures began 21 days post virus injection to allow for transduction and miR expression. Statistical analyses confirmed that the scrambled miR and nonsurgical controls groups did not significantly differ for any behavioral measures, and thus these groups were combined into a single control group for all subsequent analyses. Brains of all animals that underwent behavioral testing were subjected to post hoc immunohistochemical analysis to ensure that data was only included from animals where transduction was restricted to either the dentate gyrus or CA1 region of the hippocampus (see Supplementary Fig. 4).

### Novel object in context task

Rats were tested on their episodic contextual memory abilities using the hippocampal-dependent Novel Object in Context (NOIC) task, which was adapted from previous reports^70–72,86,87^. Briefly, each animal received a 5-min session per day in a behavioral box and the box and objects were cleaned with 10% ETOH between each animal. Rats were habituated on consecutive days to Context 1, a semi-transparent box (15in W × 24in L × 12in H) with yellow stripes, or Context 2, a gray opaque box (17in W × 17in L × 16in H), counterbalanced by group and context order. Following habituation, on day 1 of NOIC, each animal was placed in Context 1 containing a soap dispenser (Object A) and an empty mason jar (Object B) placed on diagonal, equidistant markings with ample space for the rat to circle the objects. Notably, the side the objects were located on (left or right) was counterbalanced by group. The following day (day 2 of NOIC) rats were placed in Context 2 placed in a different room with duplicates of either Object A or Object B. On the test day, day 3 of NOIC, rats were placed again in Context 2, except this time with both Object A and Object B. Depending on the duplicate objects seen on day 2 of NOIC, Object A or Object B on day 3 of NOIC was not a novel object per se, but its placement in Context 2 was novel. Untreated rats will preferentially explore the contextual novel object, an effect that would be disrupted with hippocampal inactivation^87^. On day 1 and day 3, exploration was hand-scored live by an experimenter blind to group assignments from video recordings using a ceiling Digital USB 2.0 CMOS Camera (Stöelting Co., Wood Dale, IL) and was defined as sniffing or touching the object with the nose or forepaws. Time spent exploring Novel Object in Context/(Time spent exploring Object A + Object B)] × 100 was calculated on both days and the % shift from baseline was determined by subtracting the value of day 1 from day 3.

### Novel object recognition task

NOR procedures were adapted from Beilharz et al.^88^ Briefly, a gray opaque box (17in W × 17in L × 16in H) was used as an arena and placed in a dimly lit room, achieved by pointing two desk lamps face down on opposite ends of the box. Rats were habituated to the empty arena for 10 min on the day prior to testing. The novel object and the side on which the novel object was placed were counterbalanced by the group. The test began with a 5-min familiarization phase, where rats were placed in the center of the arena, facing away from the objects, with two identical copies of the same object to explore. The objects were either two identical 12 oz. cans or two identical stemless wine glasses, counterbalanced by treatment group. The objects were chosen based on preliminary studies which determined that they are equally preferred by Sprague Dawley rats. Animals were then removed from the arena and placed in the home cage for 5 min. Meanwhile, the arena and objects were cleaned with 10% ethanol solution, and one of the objects in the arena was replaced with a different one (either the 12 oz. can or stemless wine glass, whichever the animal had not previously seen, i.e., the “novel object”). Then, the animals were again placed in the center of the arena and allowed to explore for 3 min. Time spent exploring the objects were hand-scored live from videos recorded from a ceiling Digital USB 2.0 CMOS Camera (Stöelting Co., Wood Dale, IL). Then, the Recognition index [novel object exploration (s)/[novel object exploration (s)  +  familiar object exploration (s)] was calculated for each animal, with higher values on this index indicating greater exploration of the novel object.

### Zero maze task

Rodents were tested for potential anxiety-like effects associated with injecting the βSAP97-miR AAV into either the dorsal dentate gyrus or dorsal CA1 using a Zero Maze. The Zero Maze is an elevated circular platform (63.5 cm height, 116.8 cm external diameter) with two closed zones and two open zones, all of which are equal in length. The closed zones are enclosed with 17.5 cm high walls whereas the open zones have only 3 cm high curbs. Any-Maze software (Stöelting Co., Wood Dale, IL) was used to video record the animals and analyze time spent in the open zones of the maze. Animals were placed in the maze for a single 5 min session and after each session, the apparatus was cleaned with 10% ethanol.

### Open field

Open field measures both general locomotor activity and anxiety-like behavior in the rat. Here, a large gray bin, 60 cm (L) × 56 CM (W) was used as an arena and was placed under diffuse even lighting (30 lx). A center zone (19 cm L × 17.5 cm W) was identified and marked using Any-Maze software (Stoelting Co., Wood Dale, IL) and a USB camera directly overhead connected to Any-Maze software tracked the movement of the animals. Animals were placed in the center of the box and allowed to explore the arena for 10 min, with the dependent variable being the total distance traveled (m). The apparatus was cleaned with 10% ethanol after each rat was tested.

### Statistical analysis

Paired electrophysiological recordings of eEPSC amplitudes were analyzed using paired two-tailed *T* tests. Two-sample two-tailed *T* tests were used to compare electrophysiological data across independent conditions. P-values of <0.05 were considered statistically significant (KaleidaGraph). Coefficient of Variation (CV) analysis was performed on AMPAR-eEPSCs by comparing the change in eEPSC variance with the change in mean amplitude as previously described^54,55,57,60,76,77,89,90^. CV was calculated as SD/*M* (SD = standard deviation; *M* = mean). The SD and *M* were measured, normalized and plotted for a concurrent set of stimuli from a control and its neighboring transfected cell. It has been shown both theoretically and experimentally that changes in CV^−2^ (*M*^2^/SD^2^) are independent of quantal size but vary in a predictable manner with quantal content: number of release sites *n* × presynaptic release probability, Pr; CV^−2^ = *n*Pr/(1 – Pr)^89–92^. CV analysis is presented as scatter plots with CV^−2^ values calculated for transfected cell/control cell pairs on the y-axis and mean eEPSC amplitude values of transfected/cell control cell pairs on the *x*-axis. Filled circles represent the mean ± SEM of the entire dataset. Filled circles that fall on or near the 45° (*y* = *x)* line reflect changes in quantal content while values approaching the horizontal (*y* = 1) reflect a change in quantal size. Slice immunolabeling results were replicated using slices from at least five different animals. Quantified biochemical results were replicated at least three times using independent samples. For behavioral experiments, NOIC and NOR were analyzed using a multi-factor ANOVA (Statistica Version 7; Statsoft) with the surgical group, training squad, and novel object assignment as between-subjects variables. All other behavioral tasks were analyzed using a Student’s two-tailed two-sample *T* test performed using Statistica V7. The Grubbs test for outliers was used as pre-established exclusion criteria (Prism 8). *P*-values of < 0.05 were considered statistically significant. Error bars represent standard error of the mean measurement. Sample sizes in the present study are similar to those reported in the literature^59,93^ for electrophysiological recordings of eEPSC amplitudes and, for behavioral tasks, sample size was chosen based on a priori power analyses (conducted in Statistica V7) to ensure sufficient power to detect a pre-specified effect size. For biochemical data, two-sample *T* tests were used to compare experimental conditions. Additional statistical information for all experiments performed is provided in the Source Data file.

### Reporting summary

Further information on research design is available in the Nature Research Reporting Summary linked to this article.

## Supplementary information

Supplementary Information

Reporting Summary

## Data Availability

All data supporting the findings of this study are provided within the paper and its supplementary information. A source data file is provided with this paper. All additional information will be made available upon reasonable request to the authors.
