# Supplementary material for: Schizophrenia-associated SAP97 mutations increase glutamatergic synapse strength in the dentate gyrus and impair contextual episodic memory in rats
Source: Nat Commun. 2022 Feb 10;13:798. doi: 10.1038/s41467-022-28430-5 (PMC8831576; doi:10.1038/s41467-022-28430-5)
Supplement: Supplementary file 1 — Supplementary Information [file 41467_2022_28430_MOESM1_ESM.pdf]

# SUPPLEMENTARY INFORMATION

## Schizophrenia-associated SAP97 mutations increase glutamatergic synapse strength in the dentate gyrus and impair contextual episodic memory in rats

Yuni Kay[1], Linda Tsan[1], Elizabeth A. Davis[2], Chen Tian[1], Léa Décarie-Spain[2], Anastasiia Sadybekov[3], Anna N. Pushkin[1], Vsevolod Katritch[3,4], Scott E. Kanoski[1,2] and Bruce E. Herring[1,5*]

[1]Neuroscience Graduate Program, University of Southern California, Los Angeles, CA 90089, USA.
[2]Department of Biological Sciences, Human and Evolutionary Biology Section, Dornsife College of Letters, Arts and Sciences, University of Southern California, Los Angeles, CA 90089, USA.
[3]Department of Chemistry, University of Southern California, Los Angeles, CA 90089, USA
[4]Quantitative and Computational Biology, University of Southern California, Los Angeles, CA 90089, USA.
[5]Department of Biological Sciences, Neurobiology Section, Dornsife College of Letters, Arts and Sciences, University of Southern California, Los Angeles, CA 90089, USA.

*Correspondence: bherring@usc.edu (B.E.H)

## Table of Contents

**Supplementary Fig. 1**: **βSAP97 immunolabeling in the hippocampus is specific and overlaps with MAP2 in DG granule neurons**.

**Supplementary Fig. 2**: **Supporting data for Figure 1.**

**Supplementary Fig. 3: Supporting data for Figure 2.**

**Supplementary Fig. 4: Stereotaxic injection of the AAV-βSAP97-miR into the dentate gyrus or CA1 of rats produced highly localized transduction within each region.**

**Supplementary Fig. 5: Schizophrenia-related mutations in SAP97's PDZ2 domain are predicted to impact binding to GluA1's PDZ-binding domain.**

**Supplementary Fig. 6: Supporting data for Figure 5.**

**Supplementary Table 1: Sequences of Oligonucleotides**

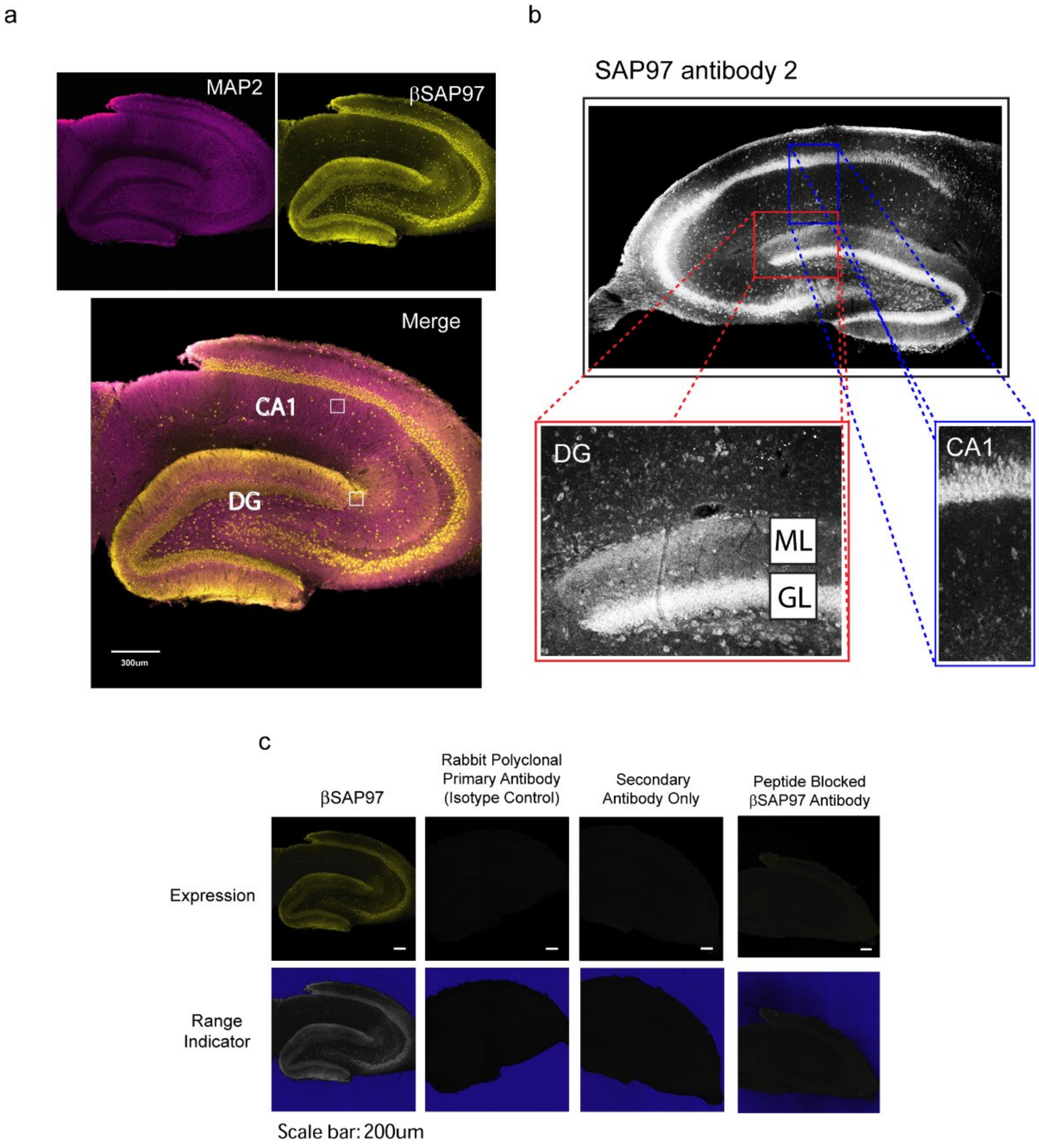


**Supplementary Fig. 1**: **βSAP97 immunolabeling in the hippocampus is specific and overlaps with MAP2 in DG granule neurons**. **a** Immunolabeling of MAP2 and βSAP97 in a P15 rat hippocampal slice. Immunolabeling of MAP2 and βSAP97 in dendrites of dentate gyrus and CA1 regions of the hippocampus shown in Fig. 1 were taken from regions shown as white squares in the merged image. **b** Immunolabeling of βSAP97 in a P15 rat hippocampal slice using a second SAP97 antibody with an epitope distinct from that used in **a** and Fig. 1a. Enlarged regions highlight SAP97 expression in DG granule neuron dendrites but not in CA1 pyramidal neuron dendrites. ML, molecular layer; GL, granule cell layer. **c** Immunolabeling with the βSAP97 antibody used in **a** and Fig. 1a, the rabbit polyclonal primary antibody as isotype control, the secondary antibody only, and the βSAP97 antibody subsequent to peptide block with corresponding range indicator images for expression levels.

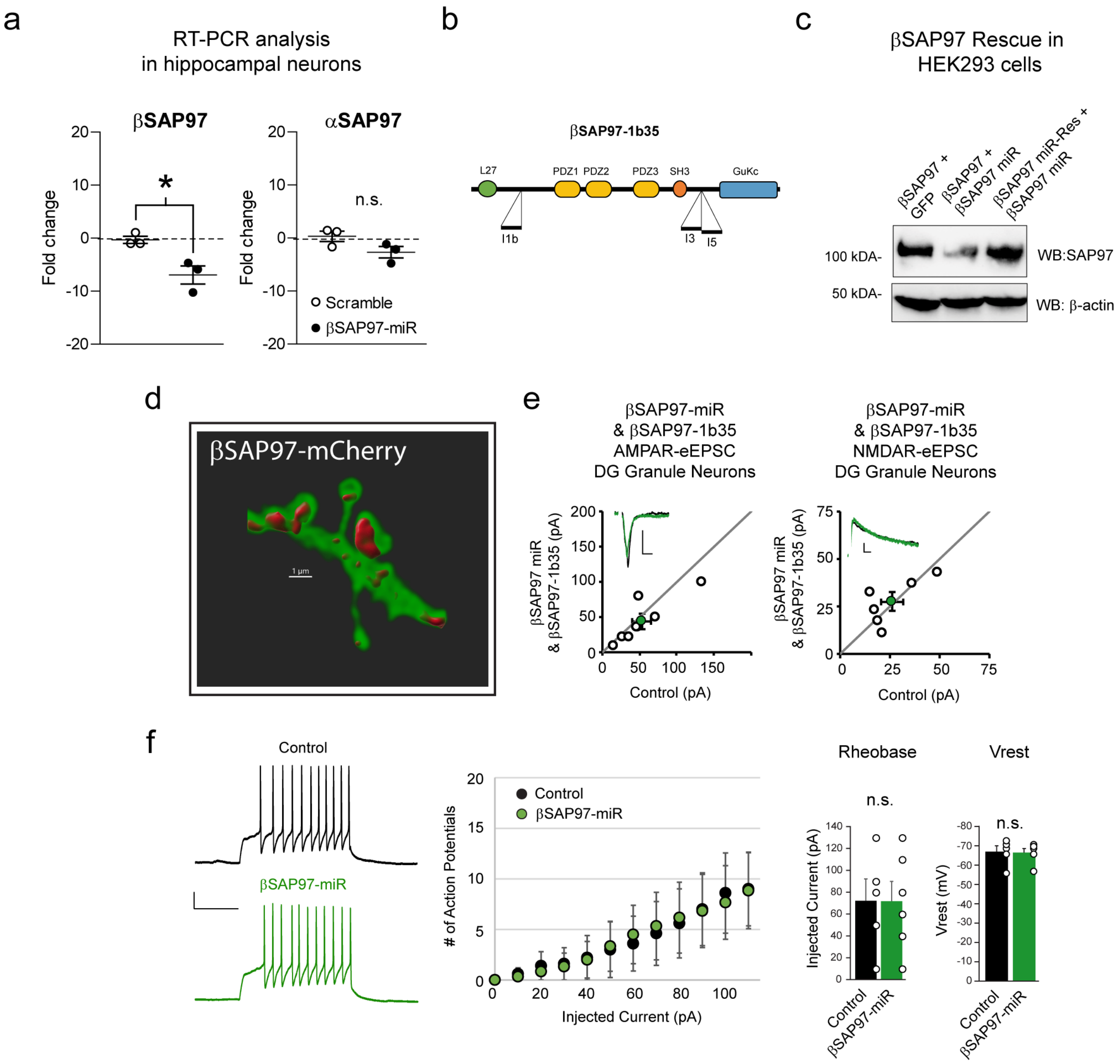

a
RT-PCR analysis
in hippocampal neurons
βSAP97
αSAP97
Fold change
*
n.s.
Scramble
βSAP97-miR
b
βSAP97-1b35
L27
PDZ1
PDZ2
PDZ3
SH3
GuKc
I1b
I3
I5
c
βSAP97 Rescue in
HEK293 cells
βSAP97 + GFP
βSAP97 + βSAP97 miR
βSAP97 miR-Res + βSAP97 miR
100 kDA-
50 kDA-
WB:SAP97
WB: β-actin
d
βSAP97-mCherry
1 μm
e
βSAP97-miR
& βSAP97-1b35
AMPAR-eEPSC
DG Granule Neurons
βSAP97-miR
& βSAP97-1b35
NMDAR-eEPSC
DG Granule Neurons
βSAP97 miR
& βSAP97-1b35 (pA)
Control (pA)
f
Control
βSAP97-miR
# of Action Potentials
Injected Current (pA)
Rheobase
Vrest
n.s.
Injected Current (pA)
Vrest (mV)

**Supplementary Fig. 2**: **Supporting data for Figure 1. a** Average βSAP97 and αSAP97 mRNA expression (±SEM) in dissociated hippocampal neurons transduced with AAVs expressing the βSAP97-miR or a scrambled miR. (Left: n = 3 scrambled wells and 3 βSAP97-miR wells, p = 0.02; Right: n = 3 scrambled wells and 3 βSAP97-miR wells, p = 0.11, two sample T-tests). **b** A cDNA expressing βSAP97 with splice inserts I1b, I3, I5 was used in the βSAP97 molecular replacement experiments in the present study. **c** Immunoblot showing insensitivity of the miR-resistant βSAP97 rescue construct to the βSAP97-miR in HEK293 cells (n = 1 experiment). Wild-type βSAP97 co-expressed with the βSAP97-miR leads to reduced expression of βSAP97 (middle lane) compared to the βSAP97 + GFP control (left lane). miR-resistant βSAP97 co-expressed with βSAP97-miR rescues this deficit (right lane) and leads to similar levels of βSAP97 expression compared to the control. **d** Imaging experiment showing synaptic localization of βSAP97-mCherry in a GFP-filled DG granule neuron in a cultured entorhino-hippocampal slice. **e** Open circles represent single pairs of control and transfected neurons, filled circles are the mean ±SEM, inset representative current traces are from control (black) and transfected (green) neurons with stimulation artifacts removed. **e** Scatter plots for βSAP97 wt rescue experiment (grey bar) in Fig. 1d, e. AMPAR-eEPSC: n = 8 pairs; NMDAR-eEPSC: n = 8 pairs. Scale bars: 20ms for AMPA, 50ms for NMDA, 20pA. **f** βSAP97-miR expression has no effect on the excitability or the resting membrane potential of DG granule neurons. (Left) Representative traces of action potentials following current injection into βSAP97-miR expressing (green) and control (black) DG granule neurons. (Center) Average number of action potentials (±SEM) produced with injected current steps of increasing amplitude in βSAP97-miR expressing and control DG granule neurons. (Right) Bar graphs showing that βSAP97-miR expression has no effect on the rheobase (Rheobase; control n = 5 neurons, βSAP97-miR n = 6 neurons, p = 0.99, two sample T-test) nor on the resting membrane potential (Vrest; control n = 5 neurons, βSAP97-miR n = 6 neurons, p = 0.89, two sample T-test). *p < 0.05; n.s., not significant. All statistical tests performed were two-tailed. Source data are provided in the Source Data file.

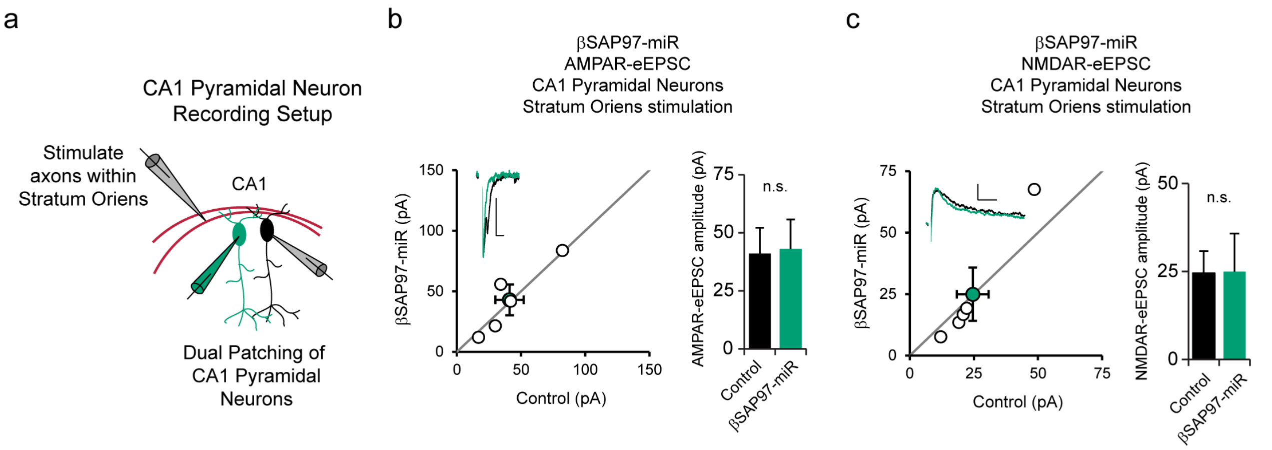


**Supplementary Fig. 3: Supporting data for Figure 2. a** Schematic representation of electrophysiological recording setup for CA1 pyramidal neurons following stimulation of axons within the stratum oriens. For **b** and **c**, open circles are single pairs of control and transfected neurons, filled circles represent the mean amplitudes (±SEM), insets show representative current traces from control (black) and βSAP97-miR transfected (green) neurons with stimulation artifacts removed. Scale bars: 20ms for AMPA, 50ms for NMDA, 20pA. Bar graphs show the average AMPAR-eEPSC and NMDAR-eEPSC amplitudes (±SEM) of CA1 pyramidal neurons expressing the βSAP97-miR (green) and control CA1 pyramidal neurons (black). βSAP97-miR expression has no effect on neither AMPAR-eEPSC amplitude (n = 5 pairs, p = 0.73, paired T-test) **b** nor NMDAR-eEPSC amplitude (n = 5 pairs, p = 0.94, paired T-test) **c** in CA1 pyramidal neurons following stimulation of axons within the stratum oriens**.** n.s., not significant. All statistical tests performed were two-tailed. Source data are provided in the Source Data file.

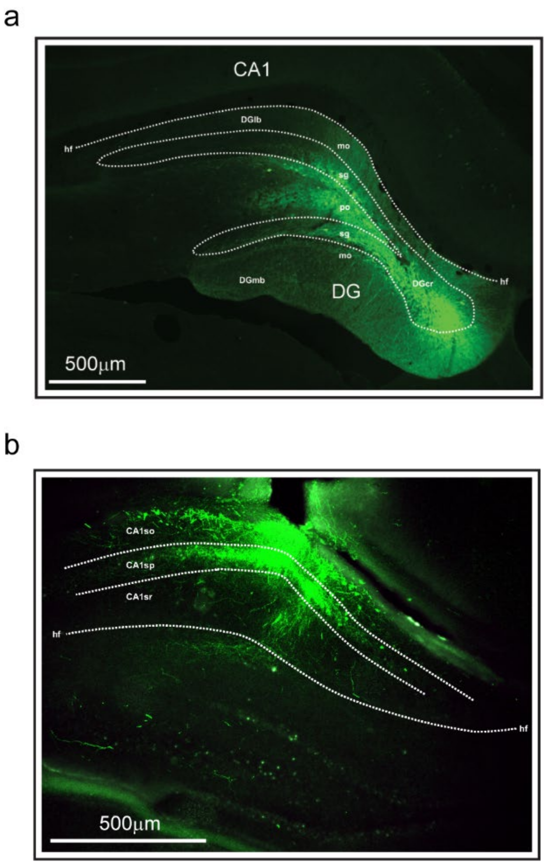


**Supplementary Fig. 4: Stereotaxic injection of the AAV-βSAP97-miR into the dentate gyrus or CA1 of rats produced highly localized transduction within each region.** Representative coronal hippocampal sections are shown that were taken from rats with AAV-βSAP97-miR injected into either the dentate gyrus **a** or CA1 region **b** subsequent to behavioral testing. Fluorescence from the GFP expressed by the AAV-βSAP97-miR construct was enhanced using immunohistochemistry. **a** Highly localized transduction of the dentate gyrus following stereotaxic injection of the AAV-βSAP97-miR. DGcr = dentate gyrus, crest; DGmb = dentate gyrus, medial blade; DGlb = dentate gyrus, lateral blade; mo = dentate gyrus, molecular layer; sg = dentate gyrus, granule cell layer; po = dentate gyrus, polymorph layer; hf = hippocampal fissure. **b** Highly localized transduction of the CA1 region following stereotaxic injection of the AAV-βSAP97-miR. CA1so = CA1, stratum oriens; CA1sp = CA1, stratum pyramidale; CA1sr = CA1, stratum radiatum.

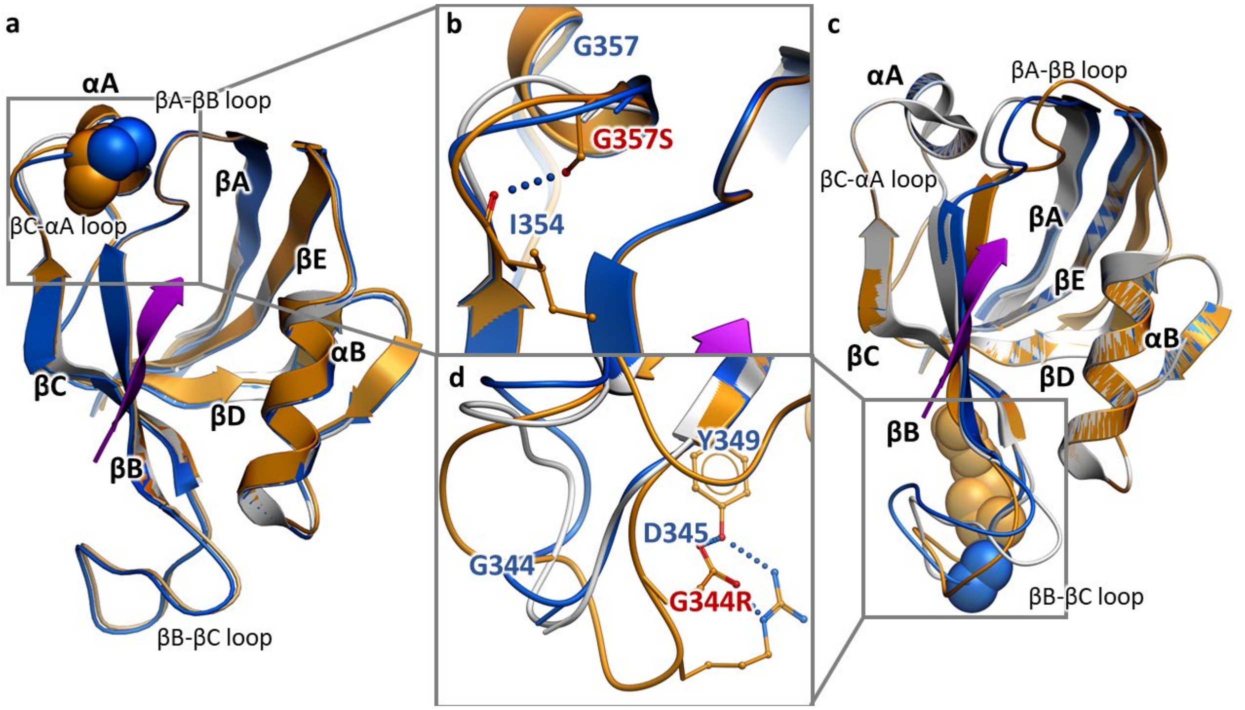


**Supplementary Fig. 5: Schizophrenia-related mutations in SAP97's PDZ2 domain are predicted to impact binding to GluA1's PDZ-binding domain. a** Superimposition of the crystal structures of SAP97's PDZ2 domain (grey) in complex with the GluA1 C-terminal peptide (magenta) (PDBID: 2G2L) and structural models of wild-type SAP97's PDZ2 domain (blue) and G357S mutant (orange) with optimized βC-αA loop. Residue G357 is shown as blue spheres and G357S is shown as orange spheres. **b** Magnified region of βC-αA loop shows the mutated G357 residue in sticks (crystal structure in white, wild-type model in blue and model of G344R mutant in orange). A hydrogen bond is shown as a blue dotted line. Our modeling showed that the mutation G357S results in the formation of a hydrogen bond between the hydroxyl group of S357 and the carbonyl oxygen of I354. Substitution of flexible Glycine to Serine and formation of an additional hydrogen bond reduces the flexibility of the βC-αA loop. Moreover, this change will likely affect the conformational changes of βA-βB observed upon peptide binding[73], as βA-βB and βC-αA loops are located in close proximity to each other. **c** Superimposition of the crystal structures of SAP97's PDZ2 domain (grey) in complex with the GluA1 C-terminal peptide (magenta) and structural models of wild-type SAP97's PDZ2 domain (blue) and G344R mutant (orange) with optimized βB-βC loop. Residue G344 is shown as blue spheres and G344R is shown as orange spheres. **d** Magnified region of βB-βC loop shows the mutated G344 residue in sticks (crystal structure in white, wild-type model in blue and model of G344R mutant in orange). Hydrogen bonds are shown as blue dotted lines. The G344R mutation was predicted to impact the conformation of βB-βC loop, as G344 has torsion angles that are not compatible with other amino acid residues.

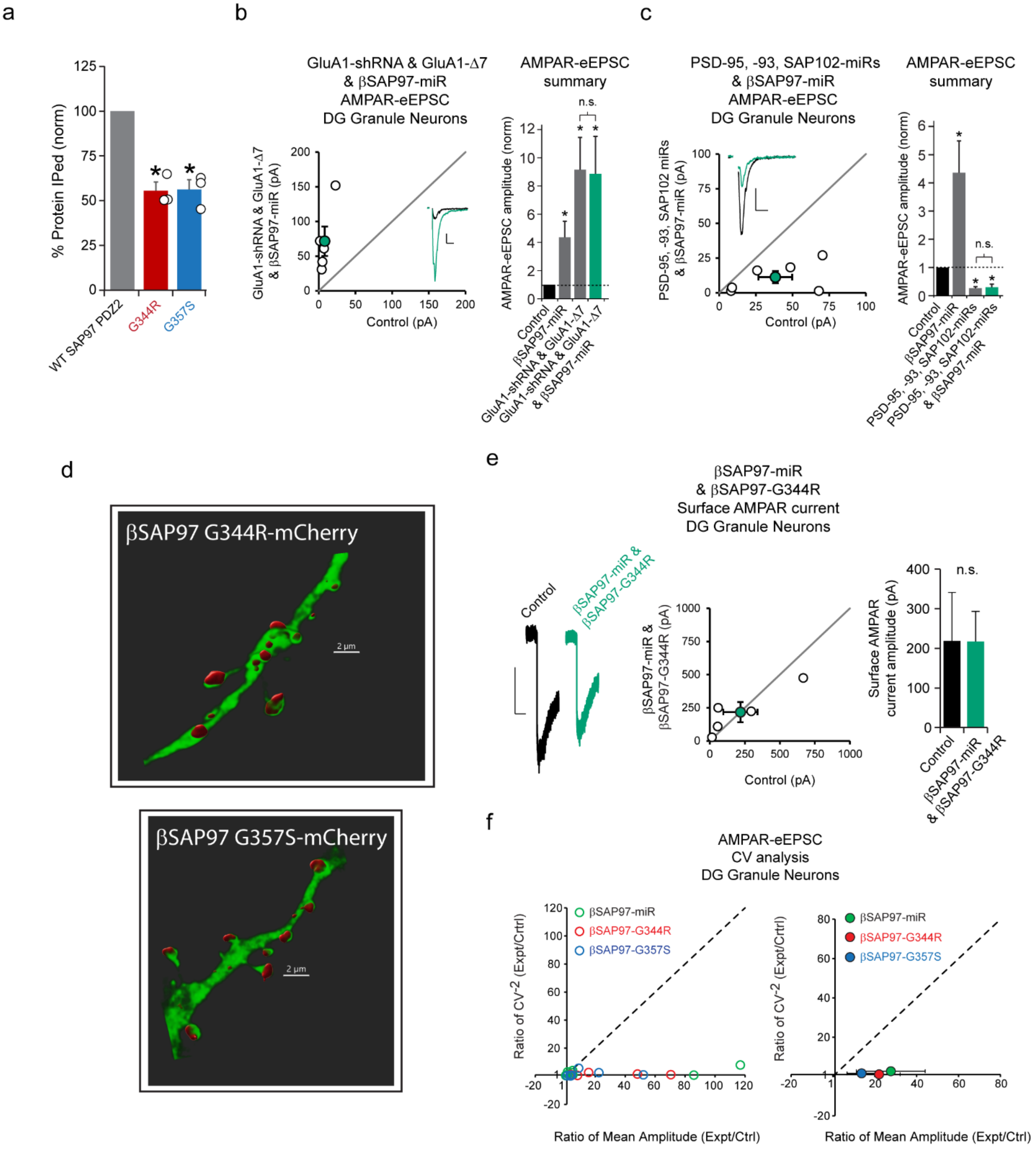
a
b
c
% Protein IPed (norm)
125
100
75
50
25
0
WT SAP97 PDZ2
G344R
G357S
GluA1-shRNA & GluA1-Δ7
& βSAP97-miR
AMPAR-eEPSC
DG Granule Neurons
GluA1-shRNA & GluA1-Δ7
& βSAP97-miR (pA)
Control (pA)
AMPAR-eEPSC
summary
AMPAR-eEPSC amplitude (norm)
n.s.
Control
βSAP97-miR
GluA1-shRNA & GluA1-Δ7
GluA1-shRNA & GluA1-Δ7
& βSAP97-miR
PSD-95, -93, SAP102-miRs
& βSAP97-miR
AMPAR-eEPSC
DG Granule Neurons
PSD-95, -93, SAP102 miRs
& βSAP97-miR (pA)
Control (pA)
AMPAR-eEPSC
summary
AMPAR-eEPSC amplitude (norm)
n.s.
Control
βSAP97-miR
PSD-95, -93, SAP102-miRs
PSD-95, -93, SAP102-miRs
& βSAP97-miR
d
βSAP97 G344R-mCherry
2 µm
βSAP97 G357S-mCherry
2 µm
e
βSAP97-miR
& βSAP97-G344R
Surface AMPAR current
DG Granule Neurons
Control
βSAP97-miR &
βSAP97-G344R
βSAP97-miR &
βSAP97-G344R (pA)
Control (pA)
Surface AMPAR
current amplitude (pA)
n.s.
Control
βSAP97-miR
& βSAP97-G344R
f
AMPAR-eEPSC
CV analysis
DG Granule Neurons
Ratio of CV$^{-2}$ (Expt/Crtrl)
βSAP97-miR
βSAP97-G344R
βSAP97-G357S
Ratio of Mean Amplitude (Expt/Ctrl)
Ratio of CV$^{-2}$ (Expt/Crtrl)
βSAP97-miR
βSAP97-G344R
βSAP97-G357S
Ratio of Mean Amplitude (Expt/Ctrl)

**Supplementary Fig. 6: Supporting data for Figure 5. a** Bar graph showing total GluA1 lysate levels Co-IPed with WT SAP97 PDZ2, G344R, or G357S normalized to the wild-type. Compared to wild-type SAP97 PDZ2, G344R and G357S have significantly less interaction with GluA1 (βSAP97-G344R, n = 3 independent experiments, p = 0.0008, two sample T-test; βSAP97-G357S, n = 3 independent experiments, p = 0.001, two sample T-test). For **b** and **c**, open circles are single pairs of control and transfected neurons, filled circles represent the mean amplitudes (±SEM), insets show representative current traces from control (black) and transfected (green) neurons with stimulation artifacts removed. Scale bars: 20ms, 20pA. Bar graphs show the average AMPAR-eEPSC amplitudes (±SEM) of DG granule neurons of various experimental conditions. **b** Molecular replacement of GluA1 with GluA1-Δ7 occludes further augmentation of AMPAR-eEPSC amplitude produced by βSAP97-miR expression in DG granule neurons. Average AMPAR-eEPSC amplitudes following expression of GluA1 shRNA, GluA1-Δ7, and βSAP97-miR (green) in DG granule neurons (n = 5 pairs) is not significantly different from the average AMPAR-eEPSC amplitudes produced by in GluA1-Δ7 molecular replacement alone (grey, n = 7 pairs; see Fig. 5f, g). p = 0.47, two sample T-test. **c** Knocking down PSD-95, PSD-93, and SAP102 eliminates the synaptic augmentation produced by βSAP97-miR expression in DG granule neurons. Average AMPAR-eEPSC amplitudes following expression of the PSD-95, PSD-93, SAP102 triple MAGUK miR along with βSAP97-miR (green, n = 6 pairs) is not significantly different from the average AMPAR-eEPSC amplitudes in the PSD-95, PSD-93, SAP102 knockdown alone (grey, n = 7 pairs, see Fig. 3e, g). p = 0.56, two sample T-test. **d** Imaging experiments showing dendritic spine localization of βSAP97 G344R-mCherry (left) and βSAP97 G357S-mCherry (right) in GFP-filled DG granule neurons in entorhino-hippocampal slices. **e** Molecular replacement of βSAP97 with βSAP97 - G344R in DG granule neurons does not change surface AMPAR current amplitude. (Left) Representative current traces, scale bar: 5s, 100pA. (Middle) Scatterplot where open circles are single pairs of control and transfected neurons and the filled circle represents the mean amplitude (±SEM.) (Right) Bar graph shows average surface AMPAR current amplitudes (±SEM) of control (black) and βSAP97-miR & βSAP97-G344R expressing (green) DG granule neurons (n = 5 pairs). p = 0.98, paired T-test. **f** Coefficient of variation (CV) analysis of AMPAR-eEPSCs from pairs of control and βSAP97-miR / βSAP97-miR & βSAP97-G344R/ βSAP97-miR & βSAP97-G357S expressing DG granule neurons. $CV^{-2}$ ratios are graphed against the mean amplitude ratio for each pair. Open circles (left): green for βSAP97-miR, n = 8 pairs; red for βSAP97-miR & βSAP97-G344R, n = 7 pairs; blue for βSAP97-miR & βSAP97-G357S, n = 7 pairs. Filled circles (right): mean ± SEM for each condition. *p < 0.05; n.s., not significant. All statistical tests performed were two-tailed. Source data are provided in the Source Data

| **Supplementary Table 1. Sequences of Oligonucleotides** | |
|---|---|
| Oligonucleotide (Primer) | Sequence |
| βSAP97 cDNA cloning into pCAGGS-IRES-mCherry, FOR | ATTCGCGGCCGCTAGCGCCACCATGCCGGTCCGGAA |
| βSAP97 cDNA cloning into pCAGGS-IRES-mCherry, REV | AGGGGCGGATCCCGGGTTTCATAGCTTTTCTTTTGCCG |
| βSAP97 cDNA editing for miR susceptibility, OLE FOR | AAGCGGCCTTAGCCCTCCAGTAGAGAAATACAGGTATCAGGATGA |
| βSAP97 cDNA editing for miR susceptibility, OLE REV | CTACTGGAGGGCTAAGGCCGCTTGGCAGTGTCTC |
| βSAP97 cDNA editing for miR susceptibility, Infusion FOR | ATTCGCGGCCGCTAGCGCCACCATG |
| βSAP97 cDNA editing for miR susceptibility, Infusion REV | AGGGGCGGATCCCGGGTCATAGCTTTTCTTTTGC |
| βSAP97 G344R, OLE FOR | GAAATCAGCATATTCCTCGGGATAATAGCATCTATGTAACCAAAATAATTGAAGGAGGTG |
| βSAP97 G344R, OLE REV | ATAGATGCTATTATCCCGAGGAATATGCTGATTTCCAACACCTCCAGC |
| βSAP97 SZ-related mutations, Infusion FOR | AAAGAATTCGCGGCCGCTAGCGCCACC |
| βSAP97 SZ-related mutations, Infusion REV | GATCGCTTCTGGCTAGTTCGAAGAGAACCTGACCCTGAACTAATACTACTATTCATCA |
| βSAP97 G357S, OLE FOR | CAAAATAATTGAAGGAAGTGCAGCACATAAGGATGGCAAACTTCA |
| βSAP97 G357S, OLE REV | CCTTATGTGCTGCACTTCCTTCAATTATTTTGGTTACATAGATGCTATTATCCCCA |
| GluA1 cDNA editing for miR resistance, OLE FOR | GAAACATGATGGTATACGTAAAATAGGTTACTGGAATGAAGACGATAAATTTGTCCCCGC |
| GluA1 cDNA editing for miR resistance, OLE REV | TCATTCCAGTAACCTATTTTACGTATACCATCATGTTTCATTTCGATCACGTGG |
| GluA1 cDNA editing for miR resistance, Infusion FOR | ATTCGCGGCCGCTAGCGCCACCATGCCGTACATCTTTGCCTT |
| GluA1 cDNA editing for miR resistance, Infusion REV | AGGGGCGGATCCCGGTTACAATCCTGTGGCTCCC |
| GluA1 cDNA editing for miR resistance & c-tail truncation, Infusion FOR | ATTCGCGGCCGCTAGCGCCACC |
| GluA1 cDNA editing for miR resistance & c-tail truncation, Infusion FOR | AGGGGCGGATCCCGGTTACATCCCTGAACT |
