# Supplementary material for: Schizophrenia-associated SAP97 mutations increase glutamatergic synapse strength in the dentate gyrus and impair contextual episodic memory in rats
Source: Nat Commun. 2022 Feb 10;13:798. doi: 10.1038/s41467-022-28430-5 (PMC8831576; doi:10.1038/s41467-022-28430-5)
Supplement: Supplementary file 2 — Reporting Summary [file 41467_2022_28430_MOESM2_ESM.pdf]

nature research

Corresponding author(s): Bruce E. Herring
Last updated by author(s): Dec 2, 2021

Reporting Summary

Nature Research wishes to improve the reproducibility of the work that we publish. This form provides structure for consistency and transparency in reporting. For further information on Nature Research policies, see our Editorial Policies and the Editorial Policy Checklist.

Statistics

For all statistical analyses, confirm that the following items are present in the figure legend, table legend, main text, or Methods section.

n/a	Confirmed	
☐	☒	The exact sample size (*n*) for each experimental group/condition, given as a discrete number and unit of measurement
☐	☒	A statement on whether measurements were taken from distinct samples or whether the same sample was measured repeatedly
☐	☒	The statistical test(s) used AND whether they are one- or two-sided<br>*Only common tests should be described solely by name; describe more complex techniques in the Methods section.*
☒	☐	A description of all covariates tested
☒	☐	A description of any assumptions or corrections, such as tests of normality and adjustment for multiple comparisons
☐	☒	A full description of the statistical parameters including central tendency (e.g. means) or other basic estimates (e.g. regression coefficient) AND variation (e.g. standard deviation) or associated estimates of uncertainty (e.g. confidence intervals)
☐	☒	For null hypothesis testing, the test statistic (e.g. *F*, *t*, *r*) with confidence intervals, effect sizes, degrees of freedom and *P* value noted<br>*Give P values as exact values whenever suitable.*
☒	☐	For Bayesian analysis, information on the choice of priors and Markov chain Monte Carlo settings
☒	☐	For hierarchical and complex designs, identification of the appropriate level for tests and full reporting of outcomes
☐	☒	Estimates of effect sizes (e.g. Cohen's *d*, Pearson's *r*), indicating how they were calculated

Our web collection on statistics for biologists contains articles on many of the points above.

Software and code

Policy information about availability of computer code

Data collection	Igor Pro v.6.37 (wavemetrics) software was used to collect electrophysiological data. Any-Maze software was used to video record animals and analyze performance on behavioral paradigms.
Data analysis	Igor (wavemetrics) software was used to analyze the electrophysiological data. KaleidaGraph software was used for statistical analysis on electrophysiological data. CM molecular modeling software (Molsoft LLC) was used to perform the structural modeling analysis. Statistica Version 7 (Statsoft) was used for statistical analysis of behavioral data. QuantStudio 5 Design & Analysis software was used to analyze RTPCR data. Imaris image analysis software (Oxford Instruments) was used to analyze SAP97 localization.

For manuscripts utilizing custom algorithms or software that are central to the research but not yet described in published literature, software must be made available to editors and reviewers. We strongly encourage code deposition in a community repository (e.g. GitHub). See the Nature Research guidelines for submitting code & software for further information.

Data

Policy information about availability of data

All manuscripts must include a data availability statement. This statement should provide the following information, where applicable:
- Accession codes, unique identifiers, or web links for publicly available datasets
- A list of figures that have associated raw data
- A description of any restrictions on data availability

All data supporting the findings of this study are provided within the paper and its supplementary information. A source data file is provided with this paper. All additional information will be made available upon reasonable request to the authors.

Field-specific reporting

Please select the one below that is the best fit for your research. If you are not sure, read the appropriate sections before making your selection.

- [x] Life sciences
- [ ] Behavioural & social sciences
- [ ] Ecological, evolutionary & environmental sciences

For a reference copy of the document with all sections, see nature.com/documents/nr-reporting-summary-flat.pdf

Life sciences study design

All studies must disclose on these points even when the disclosure is negative.

Sample size	Sample sizes for electrophysiological experiments where chosen based on prior experience with the assays performed (effect trends emerging with ~4-5 neurons/group with significance achieved with 7-8 neurons/group) and in accordance with sample sizes published in the literature (e.g. Herring et al., Neuron, 2013; Watson et al., Nature Communications, 2021). Sample size for behavioral experiments was chosen based on a priori power analyses (conducted in Statistica V7) to ensure sufficient power to detect a pre-specified effect size.
Data exclusions	No data was excluded from analysis.
Replication	To ensure reproducibility of our electrophysiological data, paired recording data was collected from 5-10 independent pairs of cells in different slices across multiple days. Datasets achieving p-values of <0.05 were considered statistically significant. To ensure reproducibility of behavioral data, animals were divided into two cohorts of Control/Experimental animals (>/= 6 animals per group) and performance of these cohorts was assessed on different days. Behavioral datasets achieving p-values of <0.05 were considered statistically significant. Conclusions were drawn from biochemical and rtPCR experiments that were replicated with independent samples >/= 3 times.
Randomization	Assignment of brain tissue/cells and animals to experimental and control conditions was carried out at random.
Blinding	Scoring of performance on behavioral assays was performed by experimenters blind to group assignment. The fluorescence of the transfected cell in paired electrophysiological recordings precludes blinding, however the lack of experimenter control over the data obtained using this technique offers a very high level of confidence in the validity of data acquired.

Reporting for specific materials, systems and methods

We require information from authors about some types of materials, experimental systems and methods used in many studies. Here, indicate whether each material, system or method listed is relevant to your study. If you are not sure if a list item applies to your research, read the appropriate section before selecting a response.

Materials & experimental systems

n/a	Involved in the study	
☐	☒	Antibodies
☐	☒	Eukaryotic cell lines
☒	☐	Palaeontology and archaeology
☐	☒	Animals and other organisms
☒	☐	Human research participants
☒	☐	Clinical data
☒	☐	Dual use research of concern

Methods

n/a	Involved in the study	
☒	☐	ChIP-seq
☒	☐	Flow cytometry
☒	☐	MRI-based neuroimaging

Antibodies

Antibodies used	MAP2 (Sigma Aldrich Cat#4403); SAP97 (Invitrogen Cat#PA1-044 and PA1-741, Neuromab, Cat#75-030, and YenZym (custom)); IgG polyclonal isotype control antibody (Abcam Cat#171870); goat anti-rabbit Alexa Fluor 555 (Invitrogen Cat#A32732); goat anti-mouse Alexa Fluor 488 (Invitrogen Cat#A-11001); β-actin (Cell Signaling Technology, Cat#4970S); Goat anti-rabbit, HRP-linked secondary antibody (Cell Signaling Technology, Cat#7074S); horse anti-mouse, HRP-linked secondary antibody (Cell Signaling Technology, Cat#7076S); PSD-95 (Millipore, Cat#637258); PSD-93 (Millipore, Cat#618436); SAP102 (Biolegend, Cat#832004); GFP (Neuromab, Cat#75-131, Invitrogen, Cat#A-11122); FLAG (Sigma, Cat#8592)
Validation	A SAP97 antibody (Rabbit Polyclonal) was custom made by YenZym. The manufacturer validated the specificity of the custom antibody to the immunizing peptide, chosen to ensure specificity for SAP97 and not the other MAGUKs, via ELISA. The antibody was further experimentally validated in our lab via immunolabeling in hippocampal slices, which led to an identical pattern of slice immunoreactivity with multiple SAP97 antibodies against distinct epitopes.<br><br>All other antibodies used in this study are commercially available and validated by the manufacturer. Validation data and citations can be found by searching the antibody catalog numbers provided here and in the Methods section, as well as through the direct links below. All primary antibodies used in western blot analyses were further experimentally validated by appropriate band size and absence of bands in non-transfected control cells.

MAP2 (Sigma Aldrich Cat#M4403)
Mouse Monoclonal
Species Reactivity: Rat, Mouse, Chicken, Human, Bovine, Quail
https://www.sigmaaldrich.com/US/en/product/sigma/m4403?context=product

SAP97 (Invitrogen Cat#PA1-044)
Rabbit Polyclonal
Species reactivity: Human, Rat
https://www.thermofisher.com/antibody/product/SAP97-Antibody-Polyclonal/PA1-044

SAP97 (Invitrogen Cat# PA1-741)
Rabbit Polyclonal
Species Reactivity: Human, Mouse, Rat
https://www.thermofisher.com/antibody/product/SAP97-Antibody-Polyclonal/PA1-741

Neuromab, Cat#75-030, and
Mouse Monoclonal
Species Reactivity: Human, Mouse, Rat
https://neuromab.ucdavis.edu/datasheet/K64_15.pdf

Rabbit IgG polyclonal isotype control antibody (Abcam Cat#171870)
https://www.abcam.com/rabbit-igg-polyclonal-isotype-control-chip-grade-ab171870.html

β-actin (Cell Signaling Technology, Cat#4970S)
Rabbit Monoclonal
Species Reactivity: Human, Mouse, Rat, Monkey, Bovine, Pig
https://www.cellsignal.com/products/primary-antibodies/b-actin-13e5-rabbit-mab/4970

PSD-95 (Millipore, Cat#637258)
Rabbit polyclonal
Species Reactivity: Rat
https://www.emdmillipore.com/US/en/product/Anti-PSD-95-Antibody,MM_NF-AB9708

PSD-93 (Millipore, Cat#618436)
Mouse Monoclonal
Species Reactivity: Mouse, Human, Rat
https://www.emdmillipore.com/US/en/product/Anti-PSD93-Antibody-clone-N18-30,MM_NF-MABN497

SAP102 (Biolegend, Cat#832004)
Mouse Monoclonal
Species Reactivity: Mouse, Human, Rat
https://www.biolegend.com/en-us/products/hrp-anti-sap102-antibody-16668?GroupID=GROUP32

GFP (Neuromab, Cat#75-131)
Mouse Monoclonal
https://neuromab.ucdavis.edu/datasheet/N86_8.pdf

GFP (Invitrogen, Cat#A-11122)
Rabbit Polyclonal
https://www.thermofisher.com/antibody/product/GFP-Antibody-Polyclonal/A-11122

FLAG (Sigma, Cat#8592)
Mouse Monoclonal
https://www.sigmaaldrich.com/US/en/product/sigma/a8592

Secondary Antibodies:

goat anti-rabbit Alexa Fluor 555 (Invitrogen Cat#A32732)
https://www.thermofisher.com/antibody/product/Goat-anti-Rabbit-IgG-H-L-Highly-Cross-Adsorbed-Secondary-Antibody-Polyclonal/A32732

goat anti-mouse Alexa Fluor 488 (Invitrogen Cat#A-11001)
https://www.thermofisher.com/antibody/product/Goat-anti-Mouse-IgG-H-L-Cross-Adsorbed-Secondary-Antibody-Polyclonal/A-11001

Anti-rabbit, HRP-linked secondary antibody (Cell Signaling Technology, Cat#7074S)
https://www.cellsignal.com/products/secondary-antibodies/anti-rabbit-igg-hrp-linked-antibody/7074

Anti-mouse, HRP-linked secondary antibody (Cell Signaling Technology, Cat#7076S)
https://www.cellsignal.com/products/secondary-antibodies/anti-mouse-igg-hrp-linked-antibody/7076

Eukaryotic cell lines

Policy information about cell lines

Cell line source(s)	HEK293 cells (ATCC, Cat#CRL-1573) Lot#70009859; HEK293T cells (ATCC, Cat#CRL-3216) Lot#63696280
Authentication	HEK293 STR Profiling: Amelogenin: X; CSF1PO: 11,12; D13S317: 12,14; D16S539: 9,13; D5S818: 8,9; D7S820: 11,12; THO1: 7,9.3; TPOX: 11; vWA: 16,19<br>HEK293T STR Profiling: CSF1PO: 11,12; D13S317: 12,14; D16S539: 9,13; D5S818: 8,9; D7S820: 11; TH01: 7, 9.3; TPOX: 11; vWA: 16,19; Amelogenin: X<br><br>Authentication information is available at the distributor website: https://www.atcc.org/products/all/CRL-1573.aspx https://www.atcc.org/products/all/CRL-3216.aspx
Mycoplasma contamination	Tests for mycoplasma contamination were performed by ATCC using the Hoechst DNA stain (indirect) method, agar culture (direct) method, and PCR-based assay. None were detected in all three tests.
Commonly misidentified lines<br>(See ICLAC register)	No commonly misidentified cell lines were used in the study.

Animals and other organisms

Policy information about studies involving animals; ARRIVE guidelines recommended for reporting animal research

Laboratory animals	P6-P15 male and female Sprague Dawley rats (Charles River) were used for tissue preparation for slice physiology and slice immunostaining. Male Sprague dawley rats (Envigo) weighing between 300-400g (~P90) were used for behavioral experiments.
Wild animals	No wild animals were used in the study.
Field-collected samples	No field collected samples were used in the study.
Ethics oversight	Experiments were performed in accordance with NIH Guidelines for the Care and Use of Laboratory Animals, and all procedures were approved by the Institutional Animal Care and Use Committee of the University of Southern California.

Note that full information on the approval of the study protocol must also be provided in the manuscript.
